# Ice-nucleating agents in sea spray aerosol identified and quantified with a holistic multimodal freezing model

**DOI:** 10.1126/sciadv.abq6842

**Published:** 2022-11-02

**Authors:** Peter A. Alpert, Wendy P. Kilthau, Rachel E. O’Brien, Ryan C. Moffet, Mary K. Gilles, Bingbing Wang, Alexander Laskin, Josephine Y. Aller, Daniel A. Knopf

**Affiliations:** ^1^ Paul Scherrer Institute, Laboratory for Environmental Chemistry, 5232 Villigen, Switzerland.; ^2^ School of Marine and Atmospheric Sciences, Stony Brook University, Stony Brook, NY 11794, USA.; ^3^ Chemical Sciences Division, Lawrence Berkeley National Laboratory, Berkeley, CA 94720, USA.; ^4^ Department of Chemistry, College of William & Mary, Williamsburg, VA 23185, USA.; ^5^ Department of Civil and Environmental Engineering, University of Michigan, Ann Arbor, MI 48109, USA.; ^6^ Department of Chemistry, University of the Pacific, Stockton, CA 95211, USA.; ^7^ Sonoma Technology, Petaluma, CA 94954, USA.; ^8^ W. R. Wiley Environmental Molecular Sciences Laboratory, Pacific Northwest National Laboratory, Richland, WA 99352, USA.; ^9^ State Key Laboratory of Marine Environmental Science, College of Ocean and Earth Sciences, Xiamen University, Xiamen 361102, China.; ^10^ Department of Chemistry, Purdue University, West Lafayette, IN 47907, USA.

## Abstract

Sea spray aerosol (SSA) is a widely recognized important source of ice-nucleating particles (INPs) in the atmosphere. However, composition-specific identification, nucleation processes, and ice nucleation rates of SSA-INPs have not been well constrained. Microspectroscopic characterization of ambient and laboratory-generated SSA confirms that water-borne exudates from planktonic microorganisms composed of a mixture of proteinaceous and polysaccharidic compounds act as ice-nucleating agents (INAs). These data and data from previously published mesocosm and wave channel studies are subsequently used to further develop the stochastic freezing model (SFM) producing ice nucleation rate coefficients for SSA-INPs. The SFM simultaneously predicts immersion freezing and deposition and homogeneous ice nucleation by SSA particles under tropospheric conditions. Predicted INP concentrations agree with ambient and laboratory measurements. In addition, this holistic freezing model is independent of the source and exact composition of the SSA particles, making it well suited for implementation in cloud and climate models.

## Teaser

Surface seawater marine microorganisms generate proteinaceous and polysaccharidic particulates that affect cold cloud formation.

## INTRODUCTION

Sea spray aerosol (SSA) particles are emitted to the atmosphere from the ocean surface by wave breaking and bubble bursting (*1*). These particles composed of inorganic salts and associated organic matter (OM) are globally abundant and represent a constant natural source of atmospheric aerosol. Climate projections rely heavily on the representation of natural aerosol sources, including SSA particles, and how they affect aerosol and cloud radiative forcing (*2*). Quantification of their impact on cloud formation is necessary to improve climate forcing estimates and our understanding of aerosol-cloud interactions (*3*). This is especially true when considering cloud glaciation by ice-nucleating particles (INPs) in mixed-phase clouds (where supercooled water droplets and ice crystals coexist), which exert notable climate feedback (*4*–*6*). Previous work suggests that ice-nucleating agents (INAs) can be aerosolized OM in SSA derived from extracellular compounds released by planktonic microorganisms into bulk subsurface water and which accumulate in the sea surface microlayer (SML) (*7*–*29*). Although various techniques and methods have been used in previous studies of SSA-INPs, as reviewed below, INAs in SSA particles have not been unambiguously identified. Here, we report on examinations of individual SSA particles using scanning transmission x-ray microscopy coupled with near-edge x-ray absorption fine structure (STXM/NEXAFS) spectroscopy that allowed identification of unique fingerprints of microbial exudates as part of those SSA particles that nucleate ice. We present a holistic approach for predicting the freezing of SSA particles by immersion freezing (IMF), deposition ice nucleation (DIN), and homogeneous ice nucleation (HIN). Derivation of this parameterization was possible by combining our findings with data from a wealth of previous studies detailing SSA and OM composition (*30*–*34*). These studies include ice nucleation data on both ambient SSA particles and those generated by plunging water jets in two mesocosm experiments and in a laboratory breaking wave channel (LBWC) with actively growing populations of microorganisms in both natural and artificial seawater (*12*, *35*). In combination, we conclude that (i) the OM associated with SSA particles is important for ice formation under conditions relevant to cirrus and mixed-phase clouds and (ii) that SSA-INPs are exudates from planktonic microorganisms released into surface ocean waters and subsequently aerosolized.

The importance of SSA particles for atmospheric ice cloud formation is well documented (*7*–*9*, *12*–*23*, *25*–*27*, *29*, *36*–*41*). Modeling results have suggested that biogenic OM plays a global role in atmospheric ice cloud formation (*10*, *15*, *26*) via IMF (i.e., ice forms from an INP immersed in a supercooled liquid). However, major uncertainties remain, primarily because of scaling INPs and associated OM in seawater to the ambient atmosphere (*10*, *11*, *26*, *42*). Inspired by historic research (*9*, *23*), the first explicitly identified marine-derived INAs were confirmed to be intact cells and cell wall fragments from three marine phytoplankton species—*Thalassiosira pseudonana*, *Nannochloris atomus*, and *Emiliani huxleyi* (*7*, *8*, *16*). Later, Wilson *et al.* (*26*) reported that SML water froze as warm as −6°C and contained some heat-sensitive compounds likely to be biogenic that retained their freezing ability after filtration through a 0.1-μm filter. This paper also presented x-ray images of phytoplankton fragments and x-ray spectroscopy of algal exudates in the sampled water and concluded that they could be responsible for forming ice (*26*). SSA particles aerosolized from an LBWC with an actively growing mixed microorganism community were probed with STXM/NEXAFS, with scanning electron microscopy (SEM), and for concentrations of INPs (*21*). Contrary to expectations, concentrations of INPs peaked before microbial growth and coincided with aerosol populations dominated by internal mixtures of sea salt and OM (*21*). That said, fewer INPs were observed over time and during all phases of growth during a bloom of microorganisms (*21*), confounding the notion that marine organisms or exudates in SSA particles serve as INAs. Further experiments were performed with the same LBWC and in a smaller marine aerosol reference tank (MART), in which a phytoplankton bloom was artificially initiated and INP concentrations measured (*12*). Ice nucleation activity as a function of temperature, *T*, was at times enhanced while suppressed at others without a clear trend in *T* during various phytoplankton bloom phases (*12*, *43*).

Investigations of the INAs associated with SSA particles have been focused on either single ice crystal residuals (*27*, *38*–*40*), dried salt residuals (*29*), or single ambient INPs (*17*). OM associated with SSA reflects the activities of planktonic microorganisms in surface waters and can include intact microorganisms or their cell wall fragments, transparent exopolymer particles, proteinaceous matter, colloidal gels, and other water-soluble or water-insoluble organic molecules associated with the release of microorganism metabolites (*44*). The immense chemical complexity of these biogenic compounds provides a multitude of potentially suitable candidates that could nucleate ice. Cornwell *et al.* (*38*) measured INPs active at *T* = −31°C at a coastal California site and probed ice crystal residuals with single-particle mass spectrometry. They observed a small fraction of ice crystal residuals classified as SSA particles, containing elevated concentrations of marine biogenic markers. Knopf *et al.* (*45*) examined particles collected in the marine boundary layer in the eastern north Atlantic for their ability to induce IMF and DIN. INPs were identified by SEM with energy-dispersive analysis of x-rays (SEM/EDX), showing particles associated with sea salt and OM. Hiranuma *et al.* (*40*) used x-ray and electron spectromicroscopy to observe specific compounds in cloud residuals (a mix of cloud water and ice particles). A large fraction of residuals containing OM, inorganic carbon, carbonate, Na, and Mg, which are indicative of a marine source, were reported as INPs (*40*). McCluskey *et al.* (*19*) also measured ice crystal residual particle morphology and composition from the LBWC and MART experiments using electron microscopy and Raman spectroscopy identifying two major ice nucleation particle types that were either cell fragments of microorganisms or OM. Wolf *et al.* (*27*) examined the ice nucleation ability of particles aerosolized from seawater containing cultures of *Prochlorococcus marinus*. Freezing was observed for particles 200 nm in diameter only when the cells in culture were lysed. The freezing fraction profile of the lysed cells was similar to those containing amylopectin (a polysaccharide) and, to a lesser extent, aspartic acid, an amino acid (*27*). During the Carbonaceous Aerosols and Radiative Effects Study (CARES) campaign, Knopf *et al.* (*17*) identified individual ambient INPs collected over the Sacramento Valley in California using STXM/NEXAFS and computer-controlled SEM/EDX (CCSEM/EDX). Individual INPs and ambient particles from marine air (named “sample SA3” in that study) contained oxidized OM dominated by carboxyl functionalities, sea salt depleted in chlorine, carbonate, and calcium (*17*).

The nucleation efficiency of INPs is mainly quantified by the INP number concentrations using their total number normalized either to air volume (INP concentration per liter of air, [INP]), surface area (ice nucleation events per square centimeter, *n*_s_), or surface area and time (ice nucleation events per square centimeter per second, *J*_het_). Application of *n*_s_ for SSA particles has been successful in previous studies, e.g., where *n*_s_ from intact and fragmented diatom cells (*7*, *8*) agreed well with those from an LBWC and a MART, using natural seawater with an actively growing microbial community (*12*). [INP] and *n*_s_ are typically determined at water saturation, i.e., in pure and highly dilute water droplets where the solute-driven freezing point depression does not play a substantial role. When considering concentrated aqueous solutions, closer reflection of conditions of SSA particles, the sheer number of solutes with various ionic strength complicates the representation of freezing point depression for [INP] and *n*_s_. A way around this that has proved to be successful was to predict heterogeneous freezing temperatures (*46*) and *J*_het_ (*14*) using water activity, *a*_w_, making it independent of the nature of the solutes, assuming that the solute does not affect the nature of the INP. Freezing temperatures and *J*_het_ were successfully applied to different species of intact and fragmented phytoplankton cells in pure water and highly concentrated aqueous solution (*7*, *8*, *14*, *16*, *36*).

In this study, we identified, using a controlled vapor cooling–stage microscope system, the components of SSA particles that nucleated ice with STXM/NEXAFS and CCSEM/EDX techniques (*17*). The SSA particles came from phytoplankton and bacterial mesocosm experiments described by Alpert *et al.* (*35*). Building on these results, we identified single INPs with different sizes from marine mesocosm experiments using STXM/NEXAFS and observed spectroscopic fingerprints of microbial exudates as responsible for ice nucleation. We determined that INAs are exudates, with a strong contribution from carboxylic functionalities as well as calcium and mixtures of amino acids and polysaccharides. In general, amino acids and polysaccharides make up about half of the marine microbial exudates composition. We emphasize the use of NEXAFS of particles described here and in previous studies (*17*, *21*, *30*, *32*, *40*, *47*) to identify the specific compounds that nucleated ice. This allows us to infer whether those compounds were also present in non-INPs and in the water, in which the phytoplankton were cultured. Furthermore, we make use of an experimental artifact, namely, the chemical change of specific compounds induced by x-ray beam damage, which provides selective evidence of specific organic INAs. We observed ice-nucleating biogenic exudates associated with individual SSA particles and argue that in clouds, all SSA particles can serve as INPs. We provide additional support for our stochastic freezing model (SFM) (*48*, *49*) for the case of SSA particles to represent simultaneously the competition between water uptake, IMF, DIN, and HIN depending on *T* and relative humidity (RH). Last, we demonstrate the SFM approach for predicting atmospheric ice particle production from aqueous SSA and cloud droplets.

## RESULTS

### Water uptake, IMF, and DIN

**Fig. 1.**
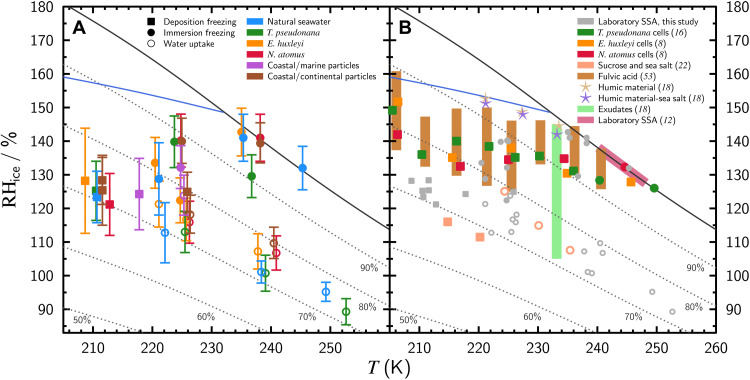
Observation of ice nucleation for laboratory-generated and field-collected particles. (**A**) Events of ice nucleation at temperature, *T*, and RH with respect to ice, RH_ice_, are plotted as filled symbols. Open symbols indicate when water uptake was observed. Colors in the legend indicate results for mesocosm experiments using natural seawater (NatSW), mesocosm experiments with different dominant phytoplankton species, and field-collected coastal particles from Shinnecock Inlet and Bayport on Long Island, NY. (**B**) The ice nucleation and water uptake data in (A) is replotted as gray symbols and compared with previous measurements (*8*, *12*, *16*, *18*, *22*, *53*), with respective symbols in the legend. Suwannee River fulvic acid (brown bars) and Suwannee River humic material (stars) were obtained from the International Humic Substances Society (*18*, *53*). In both panels, the solid black lines denote the RH with respect to water, RH = 100%. The dotted black lines indicate constant RH at 50, 60, 70, 80, and 90%. The solid blue lines show the expected homogeneous freezing limit for pure water and aqueous solution droplets 10 μm in diameter devoid of INPs.

Observations of water uptake, IMF, and DIN from laboratory-generated and field-collected particles are shown in Fig. 1. DIN induced by SSA particles was observed for all mesocosm experiments and clusters around an RH with respect to ice, RH_ice_ = 125% for *T* < 220 K. Similar values were recorded in experiments with ambient SSA particles referred to either as “coastal/marine particles” or “coastal/continental” particles. This classification was based on the sampling location and backward air mass trajectories (see Supplementary Text and fig. S1). At *T* ≥ 220 K, IMF was observed for the same particles between RH_ice_ = 122 to 143%, which is likely a result of insoluble or solid biogenic INPs in the aqueous solution. Water uptake preceded IMF shown as open circles in Fig. 1A. As detailed further in the Supplementary Text and fig. S2, this was observed at RH = 74.4 ± 3.5%, which is slightly lower than expected for NaCl particles but in line with deliquescence RH of ambient SSA particles (*37*, *50*, *51*) and those mixed with NaCl and OM (*52*). Results of DIN, IMF, and water uptake for all experiments are given in table S1. Particles with a backward air mass trajectory over land nucleated ice heterogeneously at a similar RH_ice_ compared to marine-derived particles. However, water uptake was not observed. It is likely that the examined continental particles contain less hygroscopic components compared to SSA or marine-derived particles (*24*), helping to explain why DIN was favored. In general, our laboratory-generated SSA particles and field-collected marine particles all nucleated ice heterogeneously under similar conditions.

Figure 1B compares ice nucleation results of this and previous studies. DIN associated with various phytoplankton cells shown as green, orange, and red squares (*8*, *16*) and exudates from filtered culture water shown as the light green rectangle (*18*) occur within similar conditions. It is important to note that DIN of phytoplankton cells extends to temperatures warmer than observed here for SSA particles, which may have resulted from soluble material having been washed off before observations of ice nucleation in those previous studies (*8*, *16*). Heterogeneous ice nucleation was not observed for soluble humic particles 500 nm in diameter shown as stars (*18*). However, ice nucleation was observed for humic particles in a similar range as our SSA particles and with a similar total particle surface area seen as vertical brown bars (*53*). DIN of mixed sucrose and sea salt particles occurs at lower RH_ice_ than our values, which may be due to sucrose being a solid glassy surface for ice nucleation, differences in sample surface area, or a combination of these (*22*). The surface areas for all of our samples and other sample-specific parameters are provided in table S1. In another study, dry SSA particles <1 μm in diameter were exposed to water-supersaturated conditions, and IMF was observed between *T* = 240 to 248 K and RH_ice_ = 127 to 138% (*12*). Those observations agree with our natural seawater mesocosm experiment (NatSW) for the same *T* range. In summary, our results are consistent with DIN and IMF from previous marine-derived particles consisting of mixed inorganic and biogenic material.

### X-ray spectromicroscopy of INPs

**Fig. 2.**
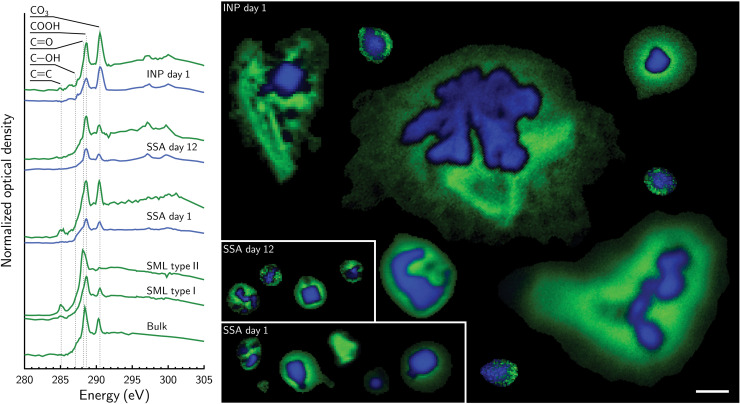
Carbon x-ray spectromicroscopy images of water-borne particles, aerosolized particles and INPs. NEXAFS spectra and corresponding x-ray component images of jet-generated SSA particles from the *T. pseudonana* mesocosm experiment (Tpseu). Blue and green lines represent spectra averaged over the blue and green regions, respectively, of particles in STXM images and normalized to their total area of OD. Only images of aerosolized particles are shown. Labeled carbon functionalities are carbonate (CO_3_), carboxyl (COOH), ketone (C═O), and carbon double bonding (C═C) appearing at 290.5, 288.6, 288.2, and 285.1 eV, respectively. Images of both INPs and those SSA particles that did not nucleate ice are included. The day after phytoplankton growth was first detected is indicated. Reference spectra of OM taken from bulk water (bulk) and the SML of axenic unialgal cultures of *T. pseudonana* are shown. The scale bar is 1 μm and applies to all images.

X-ray spectromicroscopy and single-particle identification with assessment of NEXAFS features indicate the similarity in all mesocosm experiments between of the OM associated with aerosol-borne marine INPs, non–ice-nucleating SSA particles, and OM in seawater. Figure 2 shows NEXAFS spectra and chemical maps (as false-color images) of aerosolized particles collected from a 2-week-long mesocosm experiment that contained artificial seawater and *T. pseudonana* that did and did not nucleate ice (Tpseu, see Materials and Methods). Those NEXAFS spectra are compared to spectra from bulk and SML water of unialgal and axenic cultures of *T. pseudonana*. INPs were typically large, with diameters ≥1 μm consisting of an inorganic core with an organic-rich exterior indicated by the blue and green colors, respectively. The corresponding spectra [i.e., normalized x-ray optical density (OD) as a function of x-ray energy] averaged over these respective colored regions is also shown and highlights the similarity in absorption features of both blue and green spectra corresponding to carboxyl functionalities and CO_3_. Although inorganic compounds dominated the blue regions, OM still coats the particles. These functionalities were also observed for non–ice-nucleating SSA particles generated after 1 and 12 days of phytoplankton growth.

Two distinct types of organic compounds were identified in cultures of *T. pseudonana*. In contrast, SSA particles from the Tpseu experiment showed only one specific compound. First, in the bulk and surface water of cultures, OM was present with two main absorption peaks similar to SSA particles and INPs, which we refer to as “type I” OM in Fig. 2. A second type of OM shows absorption peaks indicating C═O bonds with minor contributions of C═C bonds, which we refer to as “type II.” Although we consistently found type I OM in particles that did and did not nucleate ice, we only identified type II OM in SML water. This also suggests that type II OM may not efficiently transfer to the particle phase and therefore may not be important for ice nucleation.

**Fig. 3.**
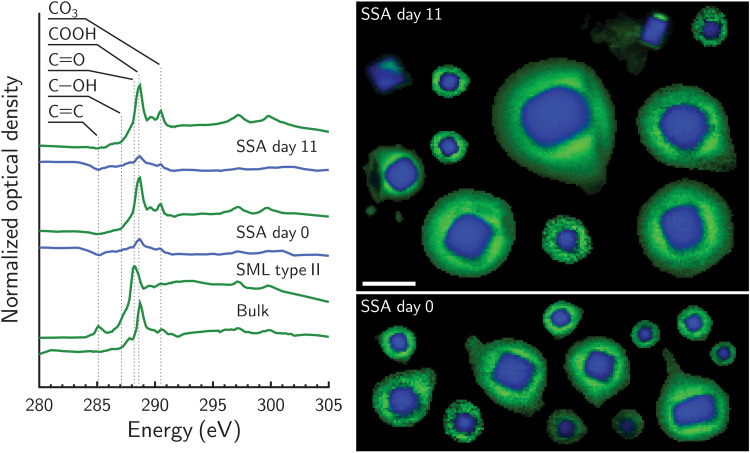
Carbon x-ray spectromicroscopy images of water-borne and aerosolized particles from NatSW. NEXAFS spectra and corresponding x-ray component images of jet-generated SSA particles from the NatSW mesocosm experiment, with a natural phytoplankton mix. Spectra of OM collected from bulk water and the SML were taken on the day the natural seawater was collected, i.e., day 0. All other labels and lines are the same as in Fig. 2.

Individual SSA particles from the NatSW mesocosm were also probed using STXM shown in Fig. 3. Type I OM was associated with SSA particles and bulk mesocosm water, but type II OM was only present in the SML. Electron micrographs, CCSEM analysis, and x-ray spectromicroscopy investigation of ice-nucleating and non–ice-nucleating SSA particles from mesocosms containing *N. atomus*, *E. huxleyi*, and a natural mix of bacteria (Natom, Ehux, and Gbac, respectively) are shown in figs. S3 to S8. Again, type I and II OM were found, but only type I OM was associated with INPs, SSA, and water. Alpert *et al.* (*35*) correlated cell counts of microorganisms, organic carbon content, and concentration of transparent exopolymer particles in water for all mesocosm experiments with aerosol particle size distributions. Those results and ours in Fig. 2 show that SSA particles had OM identical to the OM in INPs, and this observation did not depend on concentration of dissolved organic carbon, total carbon, transparent exopolymer particles, or cell numbers in the SML or bulk seawater. Although contributed by different microorganisms, OM associated with exudates similarly contributes to the composition of SSA particles and INPs.

We note that the presence and strength of the CO_3_ peaks were inconsistent, with ODs sometimes larger, smaller, or similar to the COOH peaks. While this could mean that there are highly variable distributions of CO_3_ associated with particles, this is highly unlikely given that CO_3_ should be practically undetectable being far less abundant than OM and other ions. Instead, we suggest that the CO_3_ absorption peak observed for type I OM found in INPs, non-INPs, the SML, and the bulk was the result of damage by x-ray beam exposure as discussed in Supplementary Text and shown in fig. S9. For type II OM observed in the SML, but not in SSA or bulk water, x-ray exposure caused a different damage behavior. The main peak decreased very little by comparison and coincided with a small increase in absorption due to C═C bonds. However, a CO_3_ peak did not form because of x-ray beam exposure. Unexpectedly, the spectra related to beam damage of type II OM was very similar to the spectra for xanthan gum, a high–molecular weight polysaccharide produced by *Xanthomonas campestris*, which serves as a surrogate for transparent exopolymer particles from phytoplankton exudates. In addition to the NEXAFS spectra, x-ray beam damage provides further evidence that type I and II OM are distinct and that type I is unique to natural waters, SSA particles, and INPs, making it the more likely candidate for an INA.

## DISCUSSION

### Identification of the ice nucleation organic component

**Fig. 4.**
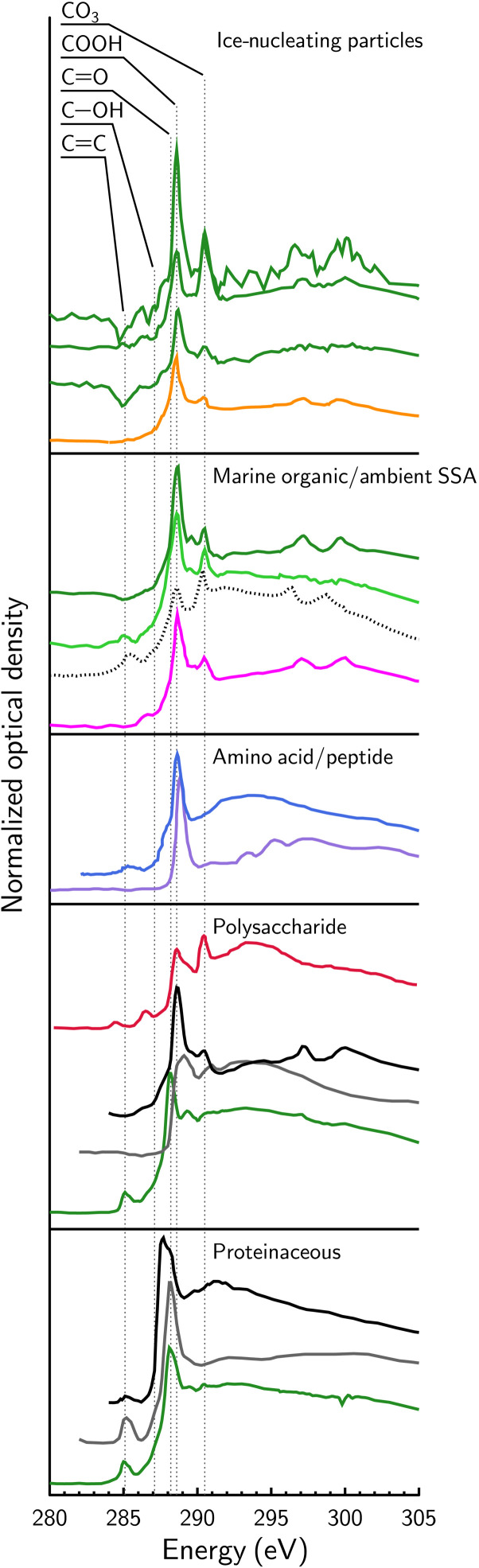
Carbon NEXAFS spectra of various types of organic and biogenic materials. Spectra labeled as “ice-nucleating particles” are shown as dark green lines from type I OM found in the Tpseu, Natom, and Ehux mesocosm experiments and the orange line from the CARES campaign (*17*). Spectra labeled as “marine organic/ambient SSA” are represented by the dark green line from type I OM in SSA particles found in the NatSW mesocosm experiment. The light green line shows data from type II OM in the SML found in the NatSW mesocosm experiment. The black dotted line shows data from the LBWC (*30*) and the pink line, marine particles collected during the CARES campaign (*31*). Spectra labeled as “amino acid/peptide” are blue (*55*) and purple (*33*). Data for spectra in the study by Hawkins and Russel (*32*) (black solid lines) reflect the presence of ambient SSA polysaccharidic and proteinaceous particles. Other colored lines show data from Braun *et al.* (*34*) (red) and Hitchcock *et al.* (*54*) (gray). We note that the dark green line labeled “polysaccharide” is from pure xanthan gum and the dark green line labeled “proteinaceous” is from the SML of the Tpseu mesocosm measured here. X-ray absorption peaks from various carbon functional groups are indicated by vertical dashed lines based on the study by Moffet *et al.* (*56*).

NEXAFS spectra of INPs, SSA particles, type I and II OM are shown in Fig. 4 in comparison with other organic compounds identified in previous studies (*30*–*34*, *54*, *55*). INPs for different mesocosm experiments have a primary x-ray absorption feature corresponding to carboxyl functionality. All “marine organic/ambient SSA” spectra have very similar features with dominant peaks indicating COOH functions and CO_3_, like type I OM observed in the current study, emphasizing its ubiquity in ambient and laboratory-generated aerosol particles. Type II OM was not common in ambient SSA or INPs. To identify the exact organic composition, we detail carbon NEXAFS spectra of different types of OM including proteins, polysaccharides, and amino acids/peptides.

The position of the most prominent peak in NEXAFS spectra occurs at 288.6 eV for ambient SSA and INPs (Fig. 4) and is most similar to the spectrum of an ambient marine INP shown by Knopf *et al.* (*17*). The blue peptide bond spectrum from the study by Polzonetti *et al.* (*55*) and the black polysaccharide spectrum from the study by Hawkins and Russel (*32*) were also similar. As previously discussed, a CO_3_ peak indicated x-ray beam damage for marine-derived OM (see fig. S9) and may indicate unique acidic polysaccharide compounds. A similar beam damage effect was observed by Braun *et al.* (*34*) for the sodium salt of alginic acid (red spectrum in Fig. 4), one of the main constituents of microbial exopolymeric substances (*57*). Other spectra for polysaccharidic and proteinaceous compounds show variability in their peak positions as shown in Fig. 4. Notice that spectra of aerosol particles from the GBac mesocosm (fig. S8) have their peak absorption at the same x-ray energy as algal exudates and aerosolized particles from all other mesocosm experiments. Although ice nucleation experiments were not carried out on SSA particles from the GBac experiment, bacterial exudates were most likely also dominated by polysaccharide and proteinaceous material (*57*), and would have nucleated ice with similar efficiency as the SSA particles from other mesocosm experiments. These results support our conclusion that microbial exudates are aerosolized and responsible for ice nucleation in ambient SSA particles.

### Cloud ice nucleation kinetics of SSA

**Fig. 5.**
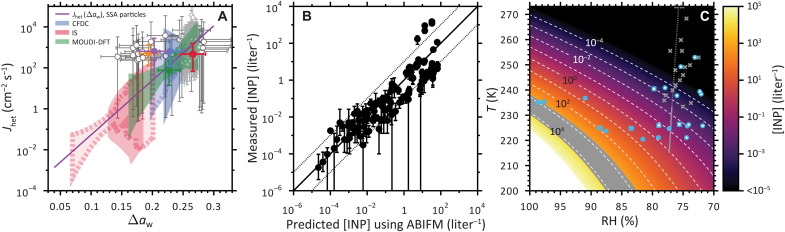
Ice nucleation kinetics and concentrations of INPs [INP] by IMF of SSA particles. (**A**) Average heterogeneous ice nucleation rate coefficients, *J*_het_, for IMF shown as circles as a function of the water activity criteria, Δ*a*_w_. Green, red, orange, and blue symbols represent the Tpseu, Natom, Ehux, and NatSW mesocosm experiments. The purple symbol represents ambient coastal/marine particles. The IMF parameterization using the water activity–based IMF model (ABIFM), log_10_ *J*_het_(Δ*a*_w_) = 26.6132Δ*a*_w_ − 3.9346, is shown as the purple line. Shadings indicate the Poisson fiducial limits of *J*_het_ derived here for experimental methods, including the CFDC, the IS, and the MOUDI-DFT (*12*). Shadings with and without a dashed outline indicate freezing data from a MART or an LBWC, respectively. Previous *J*_het_ measurements and uncertainty for IMF of particles stemming from a marine environment from Knopf *et al.* (*45*) and Cornwell *et al.* (*60*) are shown as the gray circles and diamonds, respectively. (**B**) Predicted number concentration of INPs, [INP], derived from the SFM compared with measured [INP] from previous CFDC, IS, and MOUDI-DFT experiments (*12*). The solid line represents the 1:1 line, and the dotted lines represent the 10:1 and 1:10 lines. (**C**) Predictions of [INP] as a function of RH and temperature, *T*, using *J*_het_(Δ*a*_w_) for the case of SSA particles. Blue circles and squares show data from mesocosm experiments and ambient marine/coastal particles, respectively. Filled symbols display average IMF data, and open symbols represent average water uptake data. Gray crosses represent deliquescence data of NaCl for comparison (*62*). The gray dashed line displays the expected deliquescence RH of NaCl, and the solid line represents an extrapolation to colder *T* (*51*, *62*). The gray shaded area indicates where HIN dominates.

The SFM (*15*, *36*, *48*, *49*) was newly developed to apply the data and experiments presented here and in previous work (*12*) to derive and parameterize *J*_het_ and its corresponding uncertainty. *J*_het_ for IMF is derived from our experiments, and *J*_het_ is simulated using the SFM applied to the LBWC and MART experiments that involve three different ice nucleation measurement techniques including a continuous flow diffusion chamber (CFDC), the ice spectrometer (IS), and the micro-orifice uniform deposit impactor–droplet freezing technique (MOUDI-DFT) (*12*) (Fig. 5A). Details about these techniques and the modeling procedure that mimics their operation are provided in Supplementary Text. Briefly, [INP] and *n*_s_ derived from SFM simulations are fit to previously reported [INP] and *n*_s_ data from the LBWC and MART experiments (*12*). Each experiment simulation mimics variable particle surface areas by randomly sampling of particle diameters from the reported LBWC or the MART particle size distribution (*21*, *58*).

Figure 5A shows *J*_het_ values for IMF derived from all experimental data and our parameterization. *J*_het_ is given as a function of Δ*a*_w_ (*14*, *46*), which is calculated from *a*_w_(*T*) of the aqueous solution droplet containing an INP (*a*_w_ = 1 indicates pure water) and the ice melting curve, *a*_w,ice_(*T*), resulting in Δ*a*_w_(*T*) = *a*_w_(*T*) − *a*_w,ice_(*T*). Note that Δ*a*_w_ = 0 would reflect freezing at the ice melting temperature at 273.15 K, which is not possible, and Δ*a*_w_ = 0.313 represents the homogeneous freezing limit for micrometer-sized cloud droplets (*46*, *59*). In gas-particle equilibrium, *a*_w_ of aerosol particles is assumed to equal RH (*46*). *J*_het_ exponentially increases when *T* decreases and likewise, as Δ*a*_w_ increases. To fit our SFM results to experiments, the equation log_10_ *J*_het_ = *m* Δ*a*_w_ + *b* (*14*, *36*, *49*) is used following the *a*_w_-based IMF model (ABIFM) with parameters for IMF by SSA particles as *m* = 26.6132 and *c* = −3.9346. *J*_het_ for SSA particles changed from 1.2 × 10^−2^ to 2.6 × 10^3^ cm^−2^ s^−1^ between 265 and 240 K when *a*_w_ = 1.0. This change in *J*_het_ is similar for the range of *J*_het_ determined for intact and fragmented phytoplankton cells (*14*). The shading in Fig. 5A are Poisson fiducial limits for *J*_het_ at *x* = 0.999 confidence resulting in a range of about one to three orders of magnitude. Our results are compared with that of Knopf *et al.* (*45*) who also measured *J*_het_ for IMF from ambient marine aerosol particles and found using CCSEM/EDX analysis that many INPs were SSA particles. Our results are in agreement considering all uncertainties and further support our conclusions that ambient aerosol particles contain biogenic exudates and are efficient INPs. We also compare our data with previous data from ambient SSA particles as a function of *T* by Cornwell *et al.* (*60*) having a conservative uncertainty range of two orders of magnitude shown in Fig. 5A expressed as a function of Δ*a*_w_. Overall, there is agreement between *J*_het_ determined from our experiments and from those of previous laboratory and ambient studies (*12*, *38*, *45*). Corresponding SFM calculations of *n*_s_ values are shown in fig. S10. We also derived *J*_het_ describing DIN using our experimental results and SFM simulations shown in fig. S11. For DIN by SSA particles, the same equation for *J*_het_ is used with parameters *m* = 8.2350 and *c* = 0.0559. Previous data from Knopf *et al.* (*45*) for DIN of marine particles from a marine environment are also in agreement with our *J*_het_ derivations considering all uncertainties as seen in fig. S11.

Our capability to represent IMF is further demonstrated in Fig. 5B, where we use our new *J*_het_ parameterization in Fig. 5A to predict [INP], which is excellent agreement with measured [INP] (*12*). Predicted [INP] was determined from the *J*_het_ SSA particle IMF parameterization defined above, and INPs were derived following [INP] = *J*_het_·*A*·*t*, where *A* is the reported surface area concentration in air by DeMott *et al.* (*12*) for each experiment and *t* = 1 min chosen to approximate nucleation time scales for all experiments. The root mean square error in [INP] is ±1.2 orders of magnitude, which we consider a good uncertainty estimate for predicting [INP]. This is a reasonable uncertainty considering that this is similar to the fiducial limits of *J*_het_ determined from Poisson statistics, i.e., the shading in Fig. 5A that ranges from about one to three orders of magnitude.

Using *J*_het_, we predict [INP] from SSA particles shown in Fig. 5C as a function of RH and *T* to emphasize the conditions for which SSA particles could influence ice formation in clouds. It is important to reiterate that the [INP] calculation in both Fig. 5 (B and C) does not require the computationally expensive SFM, but we apply only the simple relationship between parameterized *J*_het_, *A*, and *t*. We tested a hypothetical SSA particle population having a surface area concentration of *A* = 30 μm^2^ cm^−3^ (*61*) and an ice cloud formation time of *t*_nuc_ *=* 20 min. We find that [INP] can reach up to about 10^−1^ to 10^3^ liter^−1^ between 260 and 240 K when RH = 100%, implying that SSA may contribute markedly to IMF in mixed-phase clouds. Although we only predict [INP] in this exercise, a much more detailed model reflecting cloud conditions should be used for determining ice crystal concentration. We also modeled DIN, IMF, and HIN together in a SFM simulation shown in fig. S12, where SSA particles were sampled from a measured SSA particle size distribution. IMF was the dominant heterogeneous ice nucleation pathway for all simulations and yielded [INP] exceeding 10^2^ liter^−1^ in all cases. DIN was only active at *T* < 235 K contributing far fewer [INP] than IMF. Note in Fig. 5C that the range of HIN is shown as the gray shading, implying a limit of [INP] < 10^3^ liter^−1^ by IMF. For 77% < RH < 100%, we observed that IMF can still be important for heterogeneous ice nucleation as low as 220 K and potentially affect cirrus cloud formation.

To predict freezing from SSA particles for IMF, DIN, and HIN, we used the newly developed SFM to holistically explain results from our studies and those from previous studies including vastly different experimental setups that probed various scales. We parameterized *J*_het_ for DIN and IMF as a function of the thermodynamic parameter, Δ*a*_w_, representing RH and *T* valid for 62 to 100% and 208 to 266 K, respectively. It is important to note that apart from *J*_het_ for the SSA particles determined here, *J*_het_ specifically for marine microorganisms and their fragments have been previously quantified (*7*, *8*). We acknowledge that other types of unidentified marine or terrestrially derived INPs may be present in ambient marine samples, which would explain rare or episodic events (*20*). However, we are confident that our estimates are a quantitative analysis of the ice nucleation activity of background SSA particles. This parameterization follows the ABIFM, which is applicable to all experimental methods described. It covers the wide range of atmospheric conditions and considers freezing from aqueous solutions and pure water droplets. In addition, it does not require details about the inorganic/organic composition of SSA particles. Using SFM, we reproduce our results for both nucleation modes and for previous literature IMF data in pure or highly dilute aqueous droplets. The good agreement between our model and the data implies that random freezing is the major contributor for observed data scatter. Likewise, we found very little unexplained variance for other experimental uncertainties or other assumptions of available particle surface area for ice nucleation. These observations strongly support the parameterization following nucleation theory and thus provide practical insight into the nature of ice nucleation.

We emphasize the usefulness of the presented derivation of ice nucleation kinetics for not only predicting ice production in clouds but also serving a greater understanding of ice nucleation in general. The fact that the parameterization applies to water-subsaturated conditions makes it well suited to describe atmospheric ice particle production in a variety of cloud formation conditions. We apply idealized SSA size distributions (*35*) and predict [INP] on the basis of the combined influence of IMF, DIN, and HIN shown in fig. S12. [INP] can reach up to 10^3^ liter^−1^ under water-saturated and water-subsaturated conditions due to IMF and DIN for warmer and colder temperatures than 220 K, respectively. The fact that these conditions are common in mixed-phase and cirrus cloud regimes emphasizes the importance of ubiquitous SSA particles for ice formation. The x-ray spectral analyses of SSA particles collected from the field, generated in laboratory mesocosms with multiple planktonic microorganisms, and INPs provide strong evidence that all SSA particles derived from aquatic environments regardless of the specific makeup of the planktonic community can serve as INPs because they contain the same INAs. Finding no evidence to the contrary, our results simplify predictions of ice formation in air masses affected by marine sources.

## MATERIALS AND METHODS

### Phytoplankton and bacteria mesocosm experiments and cultures

Mesocosm experiments described by Alpert *et al.* (*35*) were conducted with artificial seawater in which phytoplankton species were grown for periods of 2 weeks. The mesocosm was sealed against ambient air to ensure that SSA production was influenced only by the microorganisms in the mesocosm. The phytoplankton species *T. pseudonana*, *N. atomus*, and *E. huxleyi* were used in three mesocosm experiments named Tpseu, Natom, and Ehux, respectively. Unialgal axenic cultures of *T. pseudonana*, *N. atomus*, and *E. huxleyi* were used to inoculate the Tpseu, Natom, and Ehux mesocosm experiments, respectively. These cultures were checked every few days to ensure that they remained axenic. A fourth mesocosm referenced as NatSW contained natural seawater with a native grown phytoplankton and bacterial population representing a natural mix of microorganisms. A final mesocosm experiment, named Gbac, contained natural seawater amended with organic carbon to grow the native mix of bacteria and kept in the dark to inhibit phytoplankton growth. The mesocosms were fitted with two different bubble generation methods, plunging water jets and glass frits. The size distributions of aerosol particles produced as a result of bubble bursting were measured over days (*35*). As the mesocosm experiments evolved in time, we tracked the concentration of cells, organic carbon, and transparent exopolymer particles generated by the microorganisms through the four phases of growth (*35*). Further details about bubble generation, bubble size measurements, aerosol particle hygroscopicity, mesocosm water composition, and mesocosm operation are given by Alpert *et al.* (*35*).

### Sampling laboratory-generated SSA, ambient marine aerosol, and phytoplankton exudates

Aerosolized particles from mesocosms were sampled using an SKC Sioutas cascade impactor (*63*) after size distribution measurements were completed. Substrates were placed into the impactor to collect particles for use in electron and x-ray spectromicroscopic investigations as well as ice nucleation experiments. These included hydrophobic glass slides (13 mm in diameter), silicon chips (2.3 mm by 2.3 mm), silicon nitride windows (1 mm by 1 mm silicon nitride membranes supported by a 5 mm by 5 mm silicon frame), and copper grids (2.3 mm in diameter with an amorphous carbon film and Formvar coating). Substrates were placed on the final stage of the impactor having stages with cutoff diameters >2.5, 1.0 to 2.5, 0.50 to 1.0, and 0.25 to 0.50 μm. The total surface area of particles on sample substrates was determined using length-calibrated images and image analysis software. First, the two-dimensional (2D) projected area and circle equivalent radius, *r*_ceq_, were determined for each particle. Typical particle morphologies in SEM images were a disk of OM surrounding a salt core. Therefore, particle morphology was approximated as a disk with radius *r*_ceq_ and a cylinder with a height of *r*_ceq_ and a radius of *r*_ceq_/2, resulting in the surface area of a single particle of 2π*r*_ceq_^2^. When optical microscopy or SEM images of approximately 200 particles each from mesocosm experiment Tpseu were compared, total sample surface area calculated varied less than a factor of 2. Exudate material from axenic unialgal cultures was sampled in two different ways. The first was using the surface lift-off method from Wilson *et al.* (*26*), in which a particle-free and OM-free silicon nitride window was put in contact with the culture water surface and lifted off. In this way, the surface-bound exudate material was sampled with very little salt residue. In the second method, the culture water was sprayed using a nebulizer, and the droplets that impacted silicon nitride windows quickly dried. This resulted in exudate material from bulk water. We also sampled xanthan gum particles by nebulizing a 0.1 weight % aqueous solution and impacting the aqueous solution droplets onto silicon nitride windows that quickly dried.

Ambient particles were collected from the south shore coast of Long Island with the times and locations are referred to as BP_Oct, SI_Oct, and SI_July, respectively, described in more detail in Supplementary Text and given in table S1. Backward air mass trajectories for each sampling time are also given in fig. S1. When sampling SI_July, the air spent days over the Atlantic Ocean before being sampled and was considered to be of marine origin. In BP_Oct and SI_Oct, particles were sampled from air that was mostly continental in origin. During BP_Oct and SI_July, the Sioutas impactor was mounted 7 m above the ground and connected to a pump at ground level. During SI_Oct, a flexible tube was mounted at 7 m connected to a multi-orifice uniform distribution impactor (MOUDI-II-122R) and pump at ground level. Aerosol particles were sampled approximately 20 m from breaking waves at these locations.

### CCSEM/EDX analysis

Images of aerosolized SSA particles on silicon chips, silicon nitride windows, and copper grids were acquired at the Environmental Molecular Sciences Laboratory located at Pacific Northwest National Laboratory using SEM. EDX analysis was also used to obtain elemental composition of particles. CCSEM was performed exclusively on copper grids using a Quanta 3D model, FEI Inc. electron microscope (*17*) for SSA particles from the Tpseu mesocosm experiment. For CCSEM analysis, thousands of particles and their EDX spectra were automatically detected using a scanning transmission electron microscopy detector and Genesis software for x-ray analysis (EDAX Inc.). This yielded the elemental and morphological information on a statistically significant number of particles. Representative electron micrographs EDX spectra and summary results from CCSEM are provided in figs. S3 to S5. Using a *k*-means machine learning cluster analysis (*17*), detected particles were classified on the basis of their elemental composition. Three clusters, *k* = 3, were used to characterize all particles. Clusters were named on the basis of their mean atomic fraction, with notable differences in the elements, C, O, Na, and Cl.

### STXM/NEXAFS analysis and individual INP identification

STXM/NEXAFS instrument at the 5.3.2 beamline of the Advanced Light Source was used to chemically map organic carbon functionalities (*56*) of individual ice-nucleating SSA particles, SSA particles that did not nucleate ice, exudate material in cultures, and xanthan gum (*17*). Briefly, this technique was used to image the organic carbon and inorganic contribution in particles with a 35 × 35 nm^2^ pixel size. Particles residing on copper grids or silicon nitride windows were placed into the STXM/NEXAFS vacuum chamber and irradiated with focused single-energy soft x-rays. The beam was raster scanned across a field of view, and the transmitted x-ray photons were measured. OD was calculated from the x-ray photon transmission through a blank substrate, *I*_o_, and through particles, *I*, following the Beer-Lamberts law, i.e., OD = −ln *I* / *I*_o_ (*56*). OD was calculated for each pixel to make up an OD image. Scanning the x-ray energy across the carbon *K*-edge from 280 to 320 eV and measuring OD generated a spatially resolved NEXAFS spectrum. This information was used to identify specific carbon bonding features such as carbon double bonding (C═C), hydroxyl functions (C─OH), ketone and carbonyl functions (C═O), carboxylic functions (COOH), and carbonate (CO_3_) mapped over individual particles. In addition, other elements such as K and Ca have absorption features at their *L*-edges near to the carbon *K*-edge and were identified. Damage to particles due to x-ray exposure was assessed by irradiating the same particle using a reduced x-ray dose multiple times and noting the absorption features that did or did not change as a result. Beam damage may alter intensities of peaks corresponding to C═C, COOH, and CO_3_ in individual particles. Therefore, we have used only the presence of absorption peaks and their peak x-ray energy position as a basis for comparison between the spectra.

We identified single particles that acted as INPs using optical microscopy during ice nucleation experiments as described below and investigated these INPs using STXM/NEXAFS following a previous procedure (*17*). After crystal growth, the ice was slowly sublimated while imaging at high magnification. The same sample of particles was taken to the x-ray microscope, and INPs were relocated manually using the image sequences. Optical and x-ray images were spatially calibrated allowing for triangulation to be used when necessary to identify particles. Here, 20 INPs were identified and imaged with STXM/NEXAFS.

### Ice nucleation experiments

Ice nucleation and water uptake occurring below or at water saturation for SSA particles were optically imaged, which allowed for discrimination between DIN and IMF events (*17*, *53*). Various samples from the Tpseu, Natom, Ehux, and NatSW mesocosm experiments were used with the two bubble generation methods and from different days of microbial growth. The ice nucleation ability of all coastal particles was investigated as well, i.e., samples from BP_Oct, SI_Oct, and SI_July. In ice nucleation experiments for both DIN and IMF, particles were cooled at a rate of 0.1 K min^−1^, and both ice formation and water uptake were observed. The frost point of the air particles were exposed to, *T*_fst_, was first adjusted and set to remain constant in a range between 205 and 255 K. Ice nucleation experiments began when RH_ice_ = 90% by adjusting the particle *T*. Particles were cooled at a rate of 0.1 K min^−1^ while imaging all particles every 0.02 K (*17*, *53*). Immediately after ice nucleated, the humidity was calibrated by warming a few degrees and finding the temperature at which the ice crystal size did not change (*17*, *53*) and thus *T* = *T*_fst_. This resulted in a small and consistent offset temperature correction between 0.1 and 0.3 K, which was applied to determine RH_ice_. Calibration yielded experimental uncertainties of Δ*T* < ±0.3 K, Δ*T*_fst_ < ±0.15 K, and ΔRH_ice_ < ±11% at 200 K and ±3% at 260 K. Multiple ice crystals nucleated at the same time in few instances, and these were considered as separate nucleation events. Frequently, ice continued to form after the first ice crystals were imaged; however, these were discarded since the water vapor pressure may not have been uniform when multiple ice crystals were present in the ice nucleation cell. In total, 68 ice nucleation events were recorded in this study.

### Stochastic freezing model

We used an SFM (*36*, *48*) to aid in determining *J*_het_ from our data and data from DeMott *et al.* (*12*). The SFM is described in detail elsewhere (*15*, *36*, *48*, *49*), and its specific application is detailed in Supplementary Text. Briefly, the SFM simulates freezing while reproducing experimental conditions, i.e., isothermal or cooling rate trajectories, and includes specific treatment of the variability of particle surface area. It also determines the variability in *T* and RH_ice_ at which ice nucleation occurs and the uncertainty of calculated *J*_het_ and *n*_s_ values due to repeating simulations in a Monte Carlo approach. DeMott *et al.* (*12*) provides an extensive dataset for ice nucleation observations of aerosolized SSA particles. We simulated their results from the LBWC and MART on all days and times that involved three different experiments, which were a CFDC, the IS, and the MOUDI-DFT, and for two different aerosolization methods, which were the LBWC and the MART. To accomplish this, particle diameters were sampled from aerosol particle size distributions of the LBWC and the MART (*21*, *58*). The total particle surface area per volume of air and the total volume of air for every simulated data point were identical to those from the study by DeMott *et al.* (*12*). The variability in the modeled ice nucleation events was due to randomly sampling of freezing events from the binomial distribution and randomly sampling from a surface area distribution. The SFM can also be used for an arbitrary aerosol population, using any *J*_het_ corresponding to different particle types and different cooling rates and under different RH and *T* trajectories. This was the case when simulating IMF, DIN, and HIN shown in fig. S12.

## Supplementary Materials

### This PDF file includes:

Supplementary Text Figs. S1 to S12 Table S1 References

## Notes

View/request a protocol for this paper from *Bio-protocol*.
