# Supplementary material for: Ice-nucleating agents in sea spray aerosol identified and quantified with a holistic multimodal freezing model
Source: Sci Adv. 2022 Nov 2;8(44):eabq6842. doi: 10.1126/sciadv.abq6842 (PMC9629709; doi:10.1126/sciadv.abq6842)
Supplement: Supplementary file 1 — Supplementary Text Figs. S1 to S12 Table S1 References [file sciadv.abq6842_sm.pdf]

Science Advances
AAAS

Supplementary Materials for

Ice-nucleating agents in sea spray aerosol identified and quantified with a holistic multimodal freezing model

Peter A. Alpert *et al.*

Corresponding author: Peter A. Alpert, peter.alpert@psi.ch;
Daniel A. Knopf, daniel.knopf@stonybrook.edu

Sci. Adv. **8**, eabq6842 (2022)
DOI: 10.1126/sciadv.abq6842

This PDF file includes:

Supplementary Text
Figs. S1 to S12
Table S1
References

Supplementary Text

Air mass origin of ambient collected particles

The backward air mass trajectories shown in Fig. S1 are from the coastal sites where ambient particles were collected and were derived from the Hybrid Single-Particle Lagrangian Integrated Trajectory computer model (HYSPLIT) (*64*). The sampling site at Shinnecock Inlet on July 29, 2013, named SI-July in Table S1 clearly has a strong marine influence leading us to describe the particles in Fig. 1 of the main manuscript as a coastal/marine type. Both of the other 2 sampling sites at Bayport Beach on October 25, 2013 and Shinnecock Inlet on October 27, 2013, named BP-Oct and SI-Oct, respectively, had a stronger continental influence and are labeled as coastal/continental.

Water uptake measurements

Observations of water uptake on SSA particles from mesocosm experiments were compared with previous studies in Fig. S2. This includes ambient collected SSA particles (*50*), particles with NaCl mixed with either glucose or laminarin (*52*), and NaCl particles without any associated OM (*51*, *62*). We observed water uptake from SSA particles as low as *RH* = 66.5% and as high as *RH* = 77.5% when *T* was near 220 K. The coastal/marine particles took up water between *RH* = 76% – 80%. The deliquescence relative humidity (DRH) of NaCl particles (*51*, *62*) tend to be slightly higher than we observed for SSA particle water uptake as can be seen in Fig. S2. SSA associated with organic matter (OM) collected from the field or in the laboratory (*50*, *52*) have a lower DRH, in agreement with previous work showing that mixtures of many different types of OM cause DRH to be lower than for pure NaCl (*65*). Note that the data indicated in blue in Fig. S2 is for ambient SSA particles with an average organic volume fraction of 0.21, and the data indicated in red are for a range of data with organic to inorganic mass fractions ranging from 1:1 to 1:3. Data from our previous mesocosm experiments (*35*) show that the organic fraction in SSA particles (green symbols) caused a decrease in the *RH* at which water uptake occurs.

Deposition ice nucleation (DIN) and immersion freezing (IMF)

Data for DIN and IMF along with water uptake from all experiments are given in Table S1. Each entry represents a single cooling rate experiment. The name, aerosolization method, surface area of the samples, and mesocosm age of the experiments in days are included. The time for ice nucleation to occur in experiments is also given in Table S1. This is the time it took for the *T* to cool at a rate of 0.1 K min-1 from the frost point, when RH_{ice} = 100%, to the *T* when nucleation was observed.

Particle morphology and elemental composition from CCSEM/EDX for SSA generated from experiments with *T. pseudonana* (Tpseu)

Figures S3 shows the SSA particle mixing state generated by computer controlled scanning electron microscopy and energy dispersive X-ray analysis (CCSEM/EDX) of particles aerosolized from the Tpseu mesocosm, in which the diatom *T. pseudonana* was grown in artificial seawater. Figure S4 shows scanning transmission electron microscope (STEM) images of representative SSA particles aerosolized from the same mesocosm. Aerosolized particles resulted from bursting bubbles

generated by impinging water jets and aeration through glass frits (*35*). Elemental ratios for a statistically significant numbers of particles analyzed using CCSEM/EDX were clustered into 3 groups using a *k*-means analysis (*17*) and identified as *i*) carbon-rich, *ii*) sea salt and carbon-rich, and *iii*) sea salt. Out of the 4181 particles analyzed in the five samples presented in Fig. S4, ~ 91 % were classified as sea salt and carbon-rich sea salt having elemental ratios of Na and Cl equal to that of the seawater used in the mesocosm. About 85 % of carbon-rich particles had circle equivalent diameters < 1.0 µm, and atomic percentages of Na and Cl were 4.0 % and 1.5 %, respectively. Figure S3A shows that with mesocosm age the number of carbon-rich particles generated using jets decreased, making up 19%, 14% and 7% of the SSA particle population, on days 3, 5, and 12, respectively. While with aerosolization by frits, production of organic-rich particles increased from 1% to 4% of the total particle number concentrations, this contribution was significantly less than the increase observed with jet generated SSA particles.

In general, our SSA particle aerosolization system using plunging seawater jets and aerating frits mimics the ambient aerosol mixing state, i.e., particles dominated either by OM or sea salt, produced by breaking waves (*21*). Figure S3B shows that for the smallest diameter range (0.2 - 0.4 µm), carbon-rich particles aerosolized in experiment Tpseu made up ~ 37% of the total particle numbers. This is in agreement with previous literature where 20 - 40% of SSA particles between 0.18 - 0.32 µm in diameter generated from a laboratory breaking wave channel (LBWC) were classified as being dominated by organic carbon (OC) (*21*). Larger particles in Tpseu, were mainly a mixture of both OM and sea salt. Although, all particle types were generated with both aerosol generation techniques. Organic-rich particles were produced in greater abundance with jets rather than frits.

Example electron micrographs of SSA particles from Tpseu are presented in Figs. S4 and S5 and show particles with an inorganic sea salt core surrounded by a “puddle” of OM. Scanning transmission X-ray microscopy coupled with near-edge X-ray absorption fine structure (STXM/NEXAFS) spectroscopy confirmed this observation as shown in Fig. 2 in the main text and Figs. S6 - S8. In rare instances, particles appeared crystallized in a branch-like structure displayed in Fig. S4 despite having a 1:1 ratio of Na:Cl. Similar structures were also seen in marine particles sampled inland near the California coast (*17, 31*) resulting from reactions with organic acids derived from secondary OM (*66*). We suggest that organic acids from SSA (primary emitted organic matter) can also lead to these unique crystalized particles. It is important to note that over 12 days of microbial growth, the concentration of *T. pseudonana* and bacterial cells in the seawater increased over 2 orders of magnitude, consistent with increases in total organic carbon, dissolved organic carbon, and transparent exopolymer particles (*35*). Nevertheless, the OM associated with single SSA particles was always present regardless of time and aerosolization method. This implies that regardless of aerosolization method, particles are morphologically and chemically similar with respect to their dominant organic functionalities despite mesocosm age and changing composition of the underlying seawater.

SEM images of generated particles from the four other mesocosm experiments are shown in Fig. S5. Two of these mesocosms (Ehux and Natom), contained a single phytoplankton species either *Emiliania huxleyi* or *Nanochloris atomus*, respectively, in addition to bacteria. In NatSW, the mesocosm contained natural seawater and a mix of naturally occurring phytoplankton and bacteria.

In the GBac experiment, the mesocosm contained a mix of bacteria previously isolated from Great South Bay, Long Island, NY but without any phytoplankton. Figure S5 clearly shows the dominant particle morphology of an inorganic core surrounded by organic material in spite of the different species and mix of bacteria or phytoplankton in conducted mesocosm experiments.

Carbon NEXAFS and chemical morphological analysis of SSA particles

Figures S6-S8 show the results of carbon NEXAFS analysis with false color STXM images of SSA and ice nucleating particles (INPs) aerosolized during mesocosm experiments. Spectra shifted vertically for clarity, were normalized to the integrated optical density, *OD*, from 280 – 320 eV. The day indicated in labels in Figs. S6-S8 is the day after the start of mesocosm experiments when aerosolized particles were generated, and collected. For example, "Day 10" means that phytoplankton were grown for 10 days with aerosolization and particle collection commencing on day 10. Spectra of the organic content of bulk/subsurface water and the surface microlayer (SML) corresponding to the species used in a specific mesocosm are included for comparison. Two distinct types of organic spectra in SML material were identified: Type I and Type II. Type I characterized by peak absorption features at 288.6 and 290.5 eV indicating carboxyl (COOH) and carbonate (CO_3) functionalities, respectively (*56*). Type II with peaks at 285.1 eV indicating carbon-carbon double bonding (C=C) (*56*) and at 288.2 eV characteristic of ketones (C=O) or proteinaceous material (*67*). The hydroxyl functionality (C-OH) is indicated by a peak at 287.1 eV (*56*) and possibly appears as a shoulder on the COOH peak. It should be noted that the presence of C-OH can also result in X-ray absorption at 289.3 eV, the main absorption peak for both glucose and aliphatic molecules with a terminal C-OH group (*68*). For consistency, labels for carbon functionalities are as per Moffet et al. (*56*).

The false color images show regions with contributions from either OM (green) or inorganic material (blue) (*17*). When probed, calcium (orange) as well as carbon was observed in spectra of INPs. The lack of any orange color in Figs. 2 and 3 (in the main text) and Figs. S6-S8 does not necessarily indicate a lack of calcium, only that calcium was not investigated. Knopf et al. (*17*) identified calcium in INPs, however, it was not a determinant for ice nucleation as it was also common to non-INPs. Occasionally, very small particles (~ 100 nm) not detected using optical microscope were found adjacent to larger identified INPs. In these cases, both were outlined by a white dashed line (Figs. S6-S8). Overall, based on STXM/NEXAFS analyses SSA particles are rich in primary OM (organic acids) associated with sea salt. Although all particles from mesocosm experiments were derived from SSA and exudate material from microorganisms, some, but not all particles nucleated ice. Even when a strong inorganic signal from sea salt cores was detected (blue color in Figs. 2 and 3 in the main text and Figs S6-S8), OM was still clearly present. Due to the fact that fragmented and intact phytoplankton cells also serve as INPs in the DIN mode (*8*) and that all particles in experiments were coated with exudates, we conclude that exudates were the only responsible ice nucleating agent (INA) for IMF.

Exploiting beam damage for organic carbon identification

Figure S9 shows the results of beam damage due to over-exposure to the X-ray beam of the two types of OM (SML Types I and II), for the polysaccharide surrogate xanthan gum and for OM

associated with SSA generated from the NatSW mesocosm. Spectra obtained from SML Type I and SSA particles initially exhibited a strong COOH peak and secondary CO_3 peak. Increased X-ray exposure caused beam damage, characterized by the reduction of the COOH peak and a corresponding increase of the CO_3 peak. Continued damage ultimately eliminated any indication of carboxyl functionalities. This coincided with a relatively smaller increase in C=C bonding. We therefore suspect that the CO_3 peak associated with the OM is the result only of damage, i.e., irradiation of COOH functionalities resulting in decarboxylation. This was observed in a previous study of alginic acid in which the authors suspected that decarboxylation had occurred resulting in reduced functionalities C=O and C=C in the condensed phase and removal of H_2O and CO to the gas phase (*34*). These data strongly suggest that CO_3 spectral peaks are largely the result of beam damage and are not indicative of the presence of CO_3^- in seawater.

SML type II material and xanthan gum showed damage from overexposure in a different way compared to Type I material and SSA particles. With the same exposure, only a minor decrease was observed for the 288.2 eV peak and an equally minor increase was observed for the 285.1 eV peak. There was no observed CO_3 before or after X-ray exposure in these samples and further, they all indicated a very different X-ray absorption and photochemical reaction pathway.

Derivation of heterogeneous ice nucleation rate coefficients for IMF

Individual heterogeneous ice nucleation rate coefficients, J_{het} with units $cm^{-2}\ s^{-1}$, were derived from IMF data pooled from each mesocosm experiment. This means, J_{het} shown in Fig. 5A of the main text is an average of all values for a single mesocosm over all days and aerosolization methods. As the T of particles decreased at a constant rate, ice nucleation was imaged every t = 12 s. The total area in each sample, A, was estimated from optical, electron and X-ray microscopy images using image analysis software to determine the 2-D projected surface area and approximating particles as spheres. The following equation,

$$J_{het} = \frac{1}{At}, \tag{S1}$$

gives J_{het} for 1 ice nucleation event that occurs in a single cooling cycle. We repeated cooling cycles N times (resulting in N ice nucleation events), and report the average T and RH at which ice formed on a sample (Fig. 1 in the main text). Accounting for multiple experimental runs of different samples, we can write,

$$J_{het} = \frac{N}{\sum At}, \tag{S2}$$

where A is the area for each sample for the ice nucleation event. When a single sample is considered, Eq (S2) simplifies to Eq (S1). The uncertainty of J_{het} was determined from Poisson statistics (*69*), or

$$J_{\mathrm{het}}^{\mathrm{up}} = \frac{N^{\mathrm{up}}}{\sum At} \quad \text{(S3)}$$

and

$$J_{\mathrm{het}}^{\mathrm{low}} = \frac{N^{\mathrm{low}}}{\sum At}, \quad \text{(S4)}$$

where N^{up} and N^{low} are the upper and lower fiducial limits for a specific N at a confidence level x = 0.999. A table of values for N^{up} and N^{low} is available in previous literature (*69*).

Derivation of ice nucleation active site (INAS) density for IMF

Ice nucleation efficiency can be quantified in terms of a sample surface area normalization. This is also termed the ice nucleation active sites (INAS) approach, expressed in the parameter n_{s} with units cm^{-2} (*70*). In our experiments, a single ice nucleation event is observed for either DIN or IMF cooling rate cycles, and therefore we derive

$$n_{\mathrm{s}} = \frac{1}{A}\ . \quad \text{(S5)}$$

This value is time independent and is interpreted to be equal to the number of ice-active surface sites on particles as a function of *T*. As previously stated, the average *T* and *RH* of multiple ice nucleation events are reported. The error in n_{s} is estimated as upper and lower fiducial limits (analogous to Eqs (S3) and (S4)),

$$n_{\mathrm{s}}^{\mathrm{up}} = \frac{N^{\mathrm{up}}}{\sum A_i} \quad \text{(S6)}$$

and

$$n_{\mathrm{s}}^{\mathrm{low}} = \frac{N^{\mathrm{low}}}{\sum A_i}. \quad \text{(S7)}$$

The stochastic freezing model (SFM) for heterogeneous ice nucleation

SFM simulations of CFDC experiments

In CFDC experiments, the probability of freezing for individual particles is given by

$$P_{\mathrm{CFDC}} = 1 - e^{-J_{\mathrm{het}} A_{\mathrm{p}} t_{\mathrm{CFDC}}}. \quad \text{(S8)}$$

CFDC experiments were conducted at constant T and for water saturation conditions, i.e., $a_w = 1.0$, and therefore, a single SFM at a single T was acquired for a constant value of J_{het} calculated from $10^{m\Delta a_w+c}$. The CFDC residence time was $t_{CFDC} = 10$ s and used in Eq (S10) to calculate P_{CFDC} for each particle. Variable surface area per particle, A_p, was simulated by random sampling diameters of single particles from previously reported size distributions for either the LBWC or the marine aerosol reference tank (MART) (*21*). Particles > 2.4 μm in diameter were not used in CFDC simulation to mimic typical operation with an impactor to remove them in experiments (*12*). Total particle surface area per volume of air sampled by the SFM was identical to that reported in DeMott et al. (*12*). For example, a reported CFDC experiment conducted on Nov. 1, 2011 from the LBWC sampled $V_{air} = 7.5$ L of air at $T = -25.2$ °C with a flow of $q = 1.5$ L min^{-1} and a particle concentration of 30 cm^{-3}. Using t_{CFDC}, this is equal to 7.5×10^3 particles in the CFDC. In a single simulation lasting t_{CFDC}, we sampled 7.5×10^3 particles from the LBWC size distribution. This resulted in a variable total surface area for every simulation that is centered around 7.9×10^{-5} cm^2 with three times the standard deviation ranging from $7.3 - 8.5 \times 10^{-5}$ cm^2. A single simulation over t_{CFDC} samples freezing from a binomial distribution once, i.e., 1 Bernoulli trial, and success is counted as a freezing event. Simulations were repeated 2×10^4 times, and a record of the number of particles that froze was kept. This procedure was repeated for all T reported in DeMott et al. (*12*). The variability in the simulated number of frozen particles at T and RH_{ice} was used to recalculate J_{het} for each simulation and average. This was done to ensure that the average recalculated J_{het} was equivalent to J_{het} used in Eq (S8) and to derive average fiducial limits of J_{het}. Additionally, the record of frozen particles was also used to recalculate values and fiducial limits of $n_s(T)$ following Eqs (S7)-(S9) in order to compare with reported $n_s(T)$ in DeMott et al. (*12*).

Following previous research (*36, 49*), we evaluated the effect of assuming that all particles had the same surface area for the derivation of J_{het} and $n_s(T)$ values. Knowing the surface area for every single particle in any experiment would be ideal; however, this is difficult and not currently feasible. In previous studies, assuming a uniform or average surface area was shown to significantly alter J_{het} and n_s values (*36, 49*), although this was not evaluated for CFDC experiments and IS or MOUDI-DFT experiment presented later. $n_s(T)$ from DeMott et al. (*12*) was derived from measured [INP] and the total particle surface area per volume of air, S_{air}, following

$$n_s^{app} = \frac{[\mathrm{INP}]}{S_{air}}, \tag{S9}$$

where n_s^{app} is the "apparent" value assuming identical surface area. Inherently, Eq (S9) assumes identical surface area per particle, since only the average total surface area is used and not a measured variability of surface area in experiments. In contrast, values of n_s^{app} from SFM simulations were calculated the same way by first determining

$$[\mathrm{INP}] = \frac{N}{V_{CFDC}}, \tag{S10}$$

where N is the simulated number of ice nucleation events and V_{CFDC} is equal to the simulated volume of air in the CFDC determined from multiplying the flowrate, $q_{CFDC} = 1.5$ L min^{-1} with

t_{CFDC}. Average values and fiducial limits of simulated $n_{\mathrm{s}}^{\mathrm{app}}$ are shown in Fig. S10a as red symbols along with experimental $n_{\mathrm{s}}^{\mathrm{app}}$ from DeMott et al. (*12*) shown as blue symbols. Considering individual particle surface area and the record of frozen particles in CFDC simulations, "actual" values were calculated as

$$n_{\mathrm{s}}^{\mathrm{act}} = \frac{N}{\sum A_{\mathrm{p}}}, \tag{S11}$$

where the total simulated surface area in the CFDC was $\sum A_{\mathrm{p}}$. Values of $n_{\mathrm{s}}^{\mathrm{act}}$ are shown as green symbols in Fig. S10a and are almost identical to $n_{\mathrm{s}}^{\mathrm{app}}$. Note that green symbols are not visible because they are almost the same as the red symbols. We conclude that under these conditions, assuming identical surface area may be valid. This was because the standard deviation stated above was determined to be small and activated INP fractions of SSA in CFDC experiments were low, on the order of 10^{-3}. We expect that particles that did not nucleate ice were a significant fraction of the total surface area, and so normalizing [INP] to total surface area is a good approximation to calculate $n_{\mathrm{s}}^{\mathrm{act}}$. In addition, we recalculated average J_{het} values using A_{p} in Eqs (S2)-(S4) instead of A, which are shown in Fig. 5A of the main text.

SFM simulations of IS experiments

The ice spectrometer (IS) experiment (*12*) was also simulated, in which water aliquots containing SSA particles were cooled at a rate of c_{r} = 1 K min^{-1}. The freezing probability is

$$P_{\mathrm{IS}} = 1 - e^{-\int J_{\mathrm{het}} A_{\mathrm{alq}} \mathrm{d}T / c_{\mathrm{r}}}, \tag{S12}$$

where dT = 0.2 K defines a simulated temperature interval and A_{alq} is the particle surface area present in a single aliquot. The application of an integral of J_{het} as a function of T was necessary due to the changing T over time. Individual values of A_{alq} were not reported in DeMott et al. (*12*) and therefore, it was randomly sampled mimicking the IS experimental procedure as follows. SSA particles generated in the LBWC or MART were filtered from air with a volume, V_{IS}, and the filter was rinsed off with washing water having a volume, V_{w}, between 5 – 10 mL (*12*). Multiple aliquots of a smaller volume, V_{alq}, between 0.05 – 0.1 mL were taken from the washing water and used in IS experiments. Simulating variable A_{alq} first required determining the average surface area in all aliquots, $\bar{A}_{\mathrm{alq}} = S_{\mathrm{air}}\, V_{\mathrm{air}}\, V_{\mathrm{alq}} / V_{\mathrm{w}}$, where S_{air} is the SSA particle surface area per volume of air for either the LBWC or MART and V_{air} is the reported air volume that was filtered (*12*). Finally, A_{alq} was randomly sampled from a lognormal distribution with ln $\bar{A}_{\mathrm{alq}}$ and σ = ln 2, as the mean and standard deviation of the corresponding normal distribution. Note that the total number of aliquots was reported to be between 24 – 48 (*12*). Therefore, we sampled the number of aliquots, N_{alq}, from a uniform distribution bounded by 24 and 48, in addition to sampling V_{alq} and V_{w}. For example, an IS experiment conducted on Nov. 8, 2011 from the LBWC filtered V_{air} = 10,500 L of air with a particle concentration of 150 cm^{-3} and S_{air} = 152 μm^2 cm^{-3} (*12*). In one simulation, random sampling resulted in N_{alq} = 41, V_{w} = 7.9 mL, V_{alq} = 0.072 mL and thus $\bar{A}_{\mathrm{alq}}$ = 0.15 cm^2. Then, A_{alq} centered on $\bar{A}_{\mathrm{alq}}$ with three times the standard deviation ranging from 0.02 – 1.1 cm^2. We repeated the simulations 10^5 times, and sampled all quantities for each repetition. Again, the record of freezing

for all aliquots for all simulations was used to recalculate average J_{het} and n_{s} values and their Poisson fiducial limits. As previously described, apparent and actual n_{s} values were calculated assuming identical surface area per aliquot or accounting for the individually sampled surface area, respectively, using Eqs (S9)-(S11). In these equations, V_{CFDC} is replaced with V_{IS}, A_{p} is replaced with A_{alq}, S_{air} was taken from IS experimental data (*12*) and simulated [INP] from the SFM for IS experiments.

In Fig. S10b, we report previous experimental values of n_{s} as blue symbols, in comparison with $n_{\mathrm{s}}^{\mathrm{act}}$ and $n_{\mathrm{s}}^{\mathrm{app}}$ as green and red symbols, respectively. Error bars on blue symbols are from DeMott et al. (*12*), while error bars on $n_{\mathrm{s}}^{\mathrm{act}}$ and $n_{\mathrm{s}}^{\mathrm{app}}$ are fiducial limits. Here, red and green symbols do not overlap and imply that assuming identical surface area per aliquot may not be valid. This is due to the range of A_{alq} being about 2 orders of magnitude, notably much larger than for CFDC simulations. A bias in the simulated results is visible at relatively warmer and colder temperatures, where $n_{\mathrm{s}}^{\mathrm{act}}$ is lower and higher, respectively, than $n_{\mathrm{s}}^{\mathrm{app}}$. In other words, assuming identical surface area tends to overestimate n_{s} at the beginning of cooling rate experiments when ice nucleation begins and underestimates n_{s} towards the end. This same apparent bias was also found in previous work (*36*, *49*). In addition, we recalculated average J_{het} values and their fiducial limits using A_{alq} in Eqs (S2)-(S4), which are shown in Fig. 5A of the main text.

SFM simulations of MOUDI-DFT experiments

SFM simulations mimicked MOUDI-DFT experiments (*12*), in which water droplets were first condensed on SSA particles impacted onto substrates and then cooled at a rate of c_{r} = 5 K min^{-1} to observe freezing. The freezing probability in the SFM is

$$P_{\mathrm{DFT}} = 1 - e^{-\int J_{\mathrm{het}} A_{\mathrm{d}} \mathrm{d}T / c_{\mathrm{r}}}, \tag{S13}$$

which is similar to Eq (S12), but using the total area inside a single droplet, A_{d}, instead of an aliquot. Experimental cooling rate trajectories were simulated in temperature intervals of, dT = 0.2 K. The faster c_{r} in these simulations compared with IS simulations resulted in shorter simulated cooling cycles of about 5 min. In MOUDI-DFT experiments, particles only from the LBWC were reported (*12*). The procedure to determine the variability of A_{d} in MOUDI-DFT experiments has been well documented by Mason et al. (*71*). Here, we detail how the SFM mimics this variability. First, the surface area on each MOUDI stage, A_{stg}^{i}, needed to be determined, where i indicates the stage number 3 – 7. Using the LBWC particle size distribution, V_{air} and S_{air} reported in DeMott et al. (*12*), and the impaction efficiency curves of the MOUDI instrument (*72*), we calculated the total particle surface area on each stage. For example, an MOUDI-DFT experiment conducted on Nov. 7-8, 2011 from the LBWC probed V_{DFT} = 28,546 L of air with a particle concentration of 175 cm^{-3} and S_{air} = 275 µm^{2} cm^{-3}. For simulations, A_{stg}^{i} for i = 3 – 7 was 3.7, 7.4, 8.9, 6.9 and 1.3 cm^{2}, respectively. The area of the region over which particles were deposited on each stage, A_{dep}^{i}, was given in Mason et al. (*71*) for i = 3 – 7 as 541.2, 541.2, 541.2, 604.8, 583.2 mm^{2} and the instrument viewing area A_{view} = 1.2 mm^{2}. As explained in detail by Mason et al. (*71*), the particle deposition across the entire MOUDI impaction area for different stages was not uniform and therefore, particle surface area in A_{view} on any stage cannot be calculated from scaling by $A_{\mathrm{view}} A_{\mathrm{stg}}^{i} / A_{\mathrm{dep}}^{i}$. Therefore, scaling

parameters for the inhomogeneity of deposited particles across all stages were $f_{\text{nu,1 mm}}$ and $f_{\text{nu,0.25-0.10 mm}}$, defined as non-uniformity factors correcting for inhomogeneous aerosol deposition at a scale of 1 mm and 0.25 – 0.10 mm, respectively (*71*). The random sampling procedure for SFM simulation first started with sampling $f_{\text{nu,1 mm}}$ and $f_{\text{nu,0.25-0.10 mm}}$ from a normal distribution using the reported standard deviation of these factors (*71*). Second, the number of droplets that condensed in the viewing area of the MOUDI-DFT, N_{d}, was sampled using normal distribution with an average of 42 droplets, a standard deviation of 14 droplets and a minimum of 5 droplets. These numbers are estimates from optical microscope images supplied in Mason et al. (*71*). Third, the diameter of droplets, D_{d}, was sampled from a uniform distribution between 65 and 135 μm, which was the reported size range in Mason et al. (*71*). Assuming that droplets were half-spheres and the SSA particle surface area in A_{view} was all distributed in droplets, the surface area in a single droplet, j, on stage i was

$$A_{\text{d}}^{j} = f_{\text{nu,1 mm}} f_{\text{nu,0.25-0.10 mm}} A_{\text{view}} \frac{A_{\text{stg}}^{i}}{A_{\text{dep}}^{i}} \frac{{D_{\text{d}}^{j}}^{2}}{\sum_{j}^{N_{\text{d}}} {D_{\text{d}}^{j}}^{2}} \;. \tag{S14}$$

For example, in a single simulation of stage 3, we sampled $f_{\text{nu,1 mm}} = 0.72$, $f_{\text{nu,0.25-0.10 mm}} = 1.03$ and $N_{\text{d}} = 29$ drops, resulting in variable A_{d} that centered on 1.7×10^{-4} cm^2 with three times the standard deviation ranging from $0.6 – 3.5 \times 10^{-4}$ cm^2. Freezing rate simulations for a cooling rate MOUDI-DFT experiment on a single stage were repeated 4×10^4 times and again for all 5 stages. The record of freezing for all droplets and stages was used to recalculate average J_{het} values and Poisson fiducial limits. $n_{\text{s}}^{\text{app}}$ and $n_{\text{s}}^{\text{act}}$ were calculated assuming identical surface area per droplet or accounting for the individually sampled surface area, respectively, using Eqs (S9)-(S11). In these equations, simulated [INP] on each stage was summed, V_{DFT} was used instead of V_{CFDC}, A_{d} was used instead of A_{p}, and S_{air} was taken from MOUDI-DFT experimental data (*12*).

In Fig. S10c, we report previous experimental values of n_{s} from MOUDI-DFT experiments as blue symbols, in comparison with $n_{\text{s}}^{\text{act}}$ and $n_{\text{s}}^{\text{app}}$ as green and red symbols, respectively, derived from the SFM simulations. Error bars on blue symbols are taken from DeMott et al. (*12*), while error bars on $n_{\text{s}}^{\text{act}}$ and $n_{\text{s}}^{\text{app}}$ are fiducial limits. Red and green symbols do not overlap meaning that assuming identical surface area per droplet may not be accurate. In addition, we recalculated average J_{het} values and their fiducial limits using A_{d} in Eq (S2)-(S4), which are shown in Fig. 5A of the main text.

Fitting SFM to IMF experimental data to derive J_{het}

Global fitting parameters m and c to calculate J_{het} as a function of Δa_{w} for SSA particles were derived from data in this study and from DeMott et al. (*12*). We used a weighted least squares fit between experimental data from DeMott et al. (*12*) and $n_{\text{s}}^{\text{app}}$. The apparent simulated value was compared because Eq (S9) was used to calculate experimentally derived n_{s} in DeMott et al. (*12*). Experimentally derived J_{het} from this study (from data using Eqs (S2)-(S4)) was fitted to J_{het} as a function of Δa_{w}. The weights were the uncertainties reported in DeMott et al. (*12*) and the fiducial limits of experimentally derived J_{het} values determined in this study. Then, m and c were adjusted

to minimize the sum of the squared residuals (RSS) between recalculated (Eq S2) and measured J_{het} from mesocosm experiments and from the coastal/marine ambient sample shown in Fig. 5 of the main text. The RSS minimization was performed on a log scale, due to J_{het} scaling logarithmically with Δa_w.

Figure S10 shows that n_s from DeMott et al. (*12*) given as blue symbols can mostly be reproduced by n_s^{app} shown as red symbols for the CFDC, IS and MOUDI-DFT experiments. Filled and open circles correspond to the LBWC and the MART, respectively. Note that only data from DeMott et al. (*12*) is shown for $a_w = 1.0$, and thus the abscissa is T. The error bars on both n_s^{app} and n_s^{act} in Fig. S10 have different length and reflect smaller or larger number of droplets that freeze in each temperature or time interval resulting in a larger or smaller error, respectively. Since these error bar lengths only consider random or systematic error (assuming identical surface area), other sources of error such as T uncertainty or instrument detection may result in slightly larger error bars if considered. It is important to note that this stochastic error depends on the number of freezing events in an interval, chosen to be the residence time in Fig. S10A and the T or t interval at which pictures of droplets or aliquots were taken as they freeze in Fig. S10B. Fundamentally, the limit of detecting freezing is when one freezing event is observed. Therefore, the largest error bars (for green and red circles) correspond to the limit of detection for freezing.

The differences between recalculated n_s^{act} and n_s^{app} from simulation are also shown in Fig. S10 and reveal the importance of representing surface area variability among individual particles, thereby, reducing the error in experiments. For the CFDC data in Fig. S10a, the difference between n_s^{act} and n_s^{app} derived from simulations is insignificant. This suggests that the size distribution from which particle diameters were sampled (*21, 58*) resulted in a narrow enough total surface area distribution inside the CFDC that the assumption of treating all particles as similar in size is valid. A similar conclusion for CFDC studies was reached when using electrical mobility size selected particles and employing a bipolar charge distribution (*36*). However, this does not mean that variability from stochastic freezing is negligible, as large error bars are present in Fig. S10a due to a low observed activated fraction. A more significant difference between n_s^{act} and $n_s^{app}$was observed for the IS and MOUDI-DFT experiments in Fig. S10b and c, respectively. For these two experiments, the RSS of n_s^{app} was one third less than the RSS for n_s^{act}. The adjusted R^2 value between simulated n_s^{app} and previous data was 0.77. The adjusted R^2 value for n_s^{act} accounting for surface area variability was 0.56, a difference of 0.21 between the RSS derived from n_s^{app}. This implies that the error associated with deriving n_s and J_{het} could be significantly reduced when accounting for surface area variability. Significant error due to assuming identical surface area per droplet was also observed previously for various mineral dust particle types using different drop freezing techniques such as cold-stage, acoustic and wind tunnel levitation experiments (*36*). In general, we highly recommend accounting for surface area variability in droplet or aliquot freezing experiments as it can reduce errors in calculating n_s or J_{het}.

Fitting SFM to DIN experimental data to derive J_{het}

Deposition ice nucleation of SSA particles observed in this study was also simulated by our SFM with the main purpose to derive J_{het} and its uncertainty, which is described in detail in previous

work (*48*). Briefly, a single simulation samples freezing from a binomial distribution with a probability for DIN as

$$P_{\mathrm{DIN}} = 1 - e^{-\int J_{\mathrm{het}} A_{\mathrm{s}} \mathrm{d}T / c_{\mathrm{r}}}, \qquad \text{(S15)}$$

where A_s is the sampled total particle area of a sample (Table S1), the experimental cooling rate in this study, c_r = 0.1 K min^{-1} and dT = 0.02 K. As described earlier, the estimated particle surface area in each sample, A, is given in Table 1. Uncertainty in surface area estimates can propagate to calculations in the ice nucleation rate coefficient (*36*). Although our estimated precision is less than a factor of 2 as described in the Methods section, surface area uncertainty is represented in the SFM by sampling A from a lognormal distribution with parameters $\mu = \ln A$ and $\sigma = 0.18$ from the corresponding normal distribution. For example, the Tpseu mesocosm experiment had $A = 1.1 \times 10^{-4}$ cm^2. A single simulation would sample A_s in a range of about 0.9 - 2.6 × 10^{-4} cm^2. This reflects a range of about a factor of 3 corresponding to 3 times the standard deviation, which is a more conservative error estimate than our measurement precision. This approach does not account for complex morphologies, e.g., rod or oblique shapes or altered particle surface area upon humidification due to soluble components. Additionally, microscopic morphological imperfections including nanopores, cavities, and cracks, are not considered in this analysis. Only the first observed DIN event is reported and so a single SFM cooling rate simulation ends as soon as 1 ice nucleation event is sampled. Simulations are repeated 2000 times, sampling A_s for each individual one. Simulations were performed for each DIN observation in Table S1, and the average T and RH_{ice} along with the standard deviation of these simulated values were derived. Fitting parameters m and c to calculate J_{het} in Eq (S15) were adjusted to minimize the RSS between the observed and predicted T and RH_{ice} for which ice nucleation occurred and weighted by the observed error. Individual observations of DIN are shown in Fig. S11A as grey crosses, where the RH_{ice} uncertainty ranges from about ± 6.5 to ± 8.7 %, depending on the T and RH_{ice}, however, the uncertainty is not shown for clarity. Only DIN data (Table S1) was used for this fit and resulted in m = 8.2350 and c = 0.0559. The RH_{ice} data scattered mainly between about 115 – 135 % and are well represented by the mean and uncertainty derived from the SFM simulations. As an exception, there are two data points at RH_{ice} = 145.3 % and 153.6 % for the Ehux mesocosm experiment at day 10 using frits generated SSA particles, which are significantly higher than the rest of the data scatter. The reason for this is not entirely clear. Except of those two data points, the rest of the data are well reproduced by our SFM, which gives confidence that DIN can be represented by $\log_{10} J_{het} = m\ \Delta a_w + c$. Data for J_{het} for marine boundary layer particles, without detailed INP surface area analysis as done in this study, are plotted in Fig. S11B for comparison. Within uncertainties, our J_{het} estimates and uncertainty bounds seen as the solid and dashed pink lines, respectively, from the SFM are in agreement with Knopf et al. (*45*). The error bars shown in Fig. S11A on the simulated data given as pink symbols are the standard deviation of the RH_{ice} at which ice was predicted to nucleate. Notice that most observations here fall within the pink error bars, which are only due to stochasticity of freezing and surface area variability. Again, this indicates that the data scatter can be largely explained by these sources of uncertainty, which leaves little unexplained error due to other factors such as T, potential non-uniform humidity field, or ice-active site variability. Therefore, our results present strong support for nucleation theory (i.e., stochastic processes) governing DIN.

Evaluating the importance of immersion freezing, deposition ice nucleation and homogeneous ice nucleation for SSA particles.

We evaluated the competition between IMF, DIN and homogeneous ice nucleation (HIN) from SSA particles using a simple box model and particle size distribution in our SFM. We sampled SSA particle dry diameters from the lognormal size distribution given in Alpert et al. (*35*) from the NatSW mesocosm experiment after 11 days of microbial growth. Then, freezing probabilities for DIN and IMF were calculated using the respective m and c fitting parameters for SSA particles derived here to calculate J_{het} in Eq (S6) using the individually sampled particle surface areas and a time interval of 10 s. Freezing was simulated using a freezing probability

$$P_{\mathrm{IMF}} = 1 - e^{-\int J_{\mathrm{het}}^{\mathrm{IMF}} A_{\mathrm{dry}} \mathrm{d}T / c_{\mathrm{r}}}, \tag{S16}$$

$$P_{\mathrm{DIN}} = 1 - e^{-\int J_{\mathrm{het}}^{\mathrm{DIN}} A_{\mathrm{dry}} \mathrm{d}T / c_{\mathrm{r}}}, \tag{S17}$$

and

$$P_{\mathrm{HIN}} = 1 - e^{-\int J_{\mathrm{hom}} V_{\mathrm{d}} \mathrm{d}T / c_{\mathrm{r}}}, \tag{S18}$$

where $J_{\mathrm{het}}^{\mathrm{IMF}}$ and $J_{\mathrm{het}}^{\mathrm{DIN}}$ are the heterogeneous ice nucleation rate coefficients for IMF and DIN, respectively, derived in this study, J_{hom} is the HIN rate coefficient parameterized as a function of Δa_{w} from previous studies (*46*, *59*), A_{dry} is the surface area corresponding to the sampled dry SSA particle, V_{d} is the liquid volume of particles, dT = 0.02 K and c_{r} = 0.1 K min^{-1}. The volume of liquid was determined from the hygroscopicity parameter, $\kappa = 1.0$ (*73*). Aerosol water volume was limited to the equivalent volume of a spherical water droplet 10 µm in diameter. Freezing was simulated, by sampling from a binomial distribution using P_{IMF}, P_{DIN} or P_{HIN}. When RH < 73%, $P_{\mathrm{IMF}} = P_{\mathrm{HIN}} = 0$. Water uptake in the SFM occurred when $RH \geq 73$ %, at which IMF and HIN was allowed and $P_{\mathrm{DIN}} = 0$. Sampling for IMF and HIN occurred simultaneously, or in other words, freezing was sampled from both binomial distributions at the same time. The number of ice nucleation events were recorded, and the concentrations of INPs were calculated from the simulated air volume of about 650 L used in the SFM. Aerosol particle concentrations were 150 cm^{-3}. Three SFMs were run with the same particle population, and using three different water vapor pressures equivalent to a frost point temperature, T_{fst}, of 210 K, 235 K and 260 K. The maximum RH was limited to 100% when the simulated air temperature exceeded the dew point. Note the values of T_{fst} correspond only to initial conditions.

The goal of this calculation is to elucidate the importance of heterogeneous ice nucleation due to SSA compared with HIN, although it does not accurately represent atmospheric ice nucleation because it lacks representation of realistic air mass trajectories, crystal growth, water vapor depletion, cloud structure and other thermodynamic and meteorological processes that influence cloud microphysics. However, it does establish the relative difference between IMF and DIN and the range of RH and T for which these pathways will dominate ice nucleation. Further study utilizing parcel or cloud-resolving model strategies would be required for a more accurate assessment.

Figure S12 shows the [INP] simulated in our SFM. At T_{fst} = 210 K, DIN results are shown as the black dotted line occurring at warmer *T* than IMF shown as the green dotted line. [INP] due to DIN reached a maximum of about 1 L^{-1}. After further cooling, *RH* > 73% and IMF (green dotted line) began yielding a maximum [INP] up to 10^2 L^{-1}, far higher than DIN. After further cooling, HIN (blue dotted line) dominated yielding [INP] far surpassing DIN and HIN. For simulations using initial T_{fst} = 235 K, a maximum of [INP] = 0.06 was predicted by DIN in our SFM. At greater T_{fst}, DIN did not occur and shows that this ice formation pathway may be only important for T < 235 K for SSA particles. IMF shown as green lines and shading always yielded higher [INP] than DIN and may be especially important for heterogeneous ice nucleation for mixed phase clouds as warm as 258 K. These SFMs imply that DIN and IMF both may explain ice crystal concentrations from SSA in the atmosphere for *T* < 235 K, however IMF should dominate at warmer *T*. Bulk water uptake and IMF always precedes HIN and may be important for cirrus or mixed-phase cloud formation between 232 K – 259 K. At 258 K and a frost point of 260 K, [INP] reached > 0.1 L-1, which is similar to commonly observed concentrations in the atmosphere under mixed-phase cloud formation (*74*, *75*), and stresses the importance of incorporating SSA as IMF INPs in cloud modeling studies (*76*). Of course, the number of ice crystals and their growth causing water vapor depletion should be taken into account when evaluating the competition between HIN and heterogeneous ice nucleation, especially for cirrus cloud formation (*77*). It is important to note that freezing in the immersion mode can occur when *RH* < 100%, meaning that parameterizations that include only IMF data acquired at water saturation or supersaturation represent an incomplete population of SSA INPs. When representing ice nucleation for various atmospheric conditions, including a variety of particle solutes and considering water subsaturated conditions, we recommend using an a_w based description of J_{het} to predict ice crystal number concentrations (*14*).

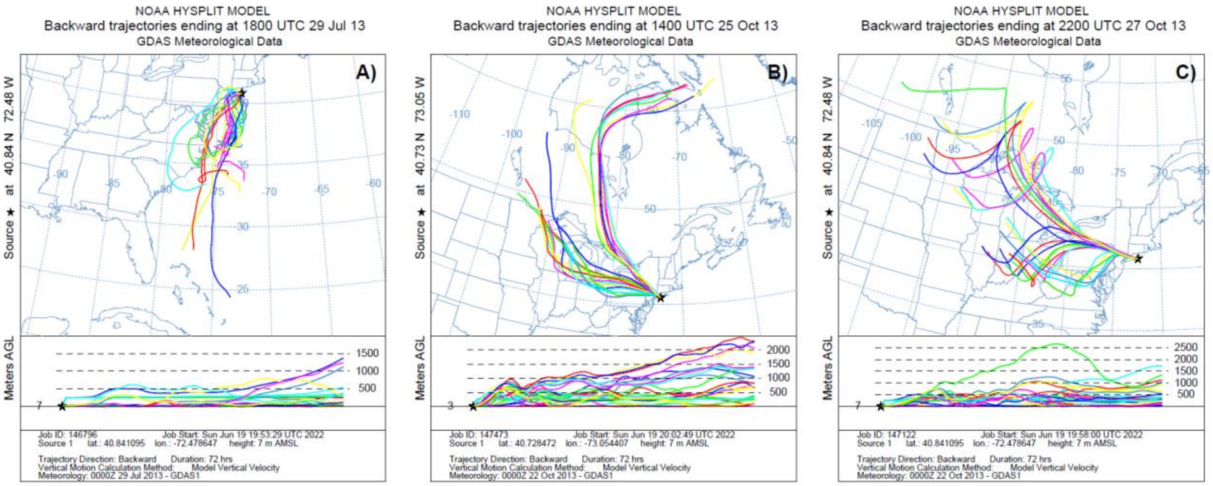

Figure S1: Backwards air mass trajectories calculated from the HYSPLIT model for ambient particle collection for the three sampling periods: A SI-July, B BP-Oct, and C SI-Oct.

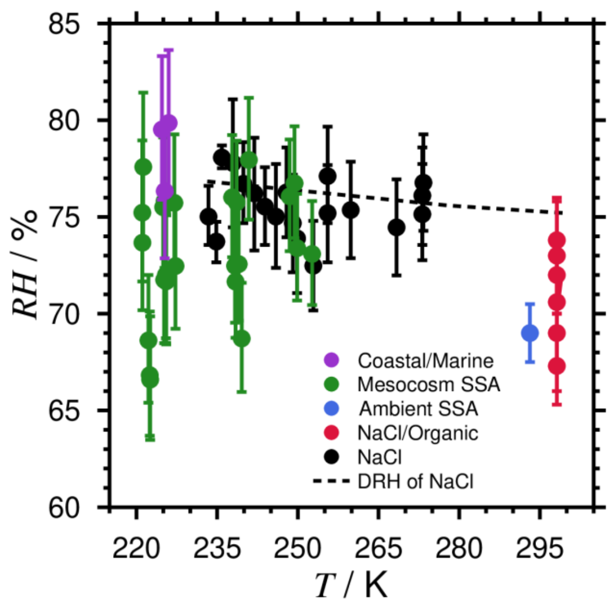

Figure S2: Observations of water uptake. SSA generated from mesocosm experiments and SSA collected from coastal/marine air are shown as green and purple symbols, respectively, from this study. Previous measurements of deliquescence relative humidity (DRH) are shown: NaCl particles (black symbols) *(51, 62)*, ambient SSA particles with an organic volume fraction of 0.21 (blue symbols) *(50)*, and NaCl particles mixed with either glucose at mass ratios of 1:1 and 1:2 or mixed with laminarin at mass ratios of 1:1, 1:2 and 1:3 (red symbols) *(52)*. The dashed line is the predicted DRH for NaCl *(51, 62)*.

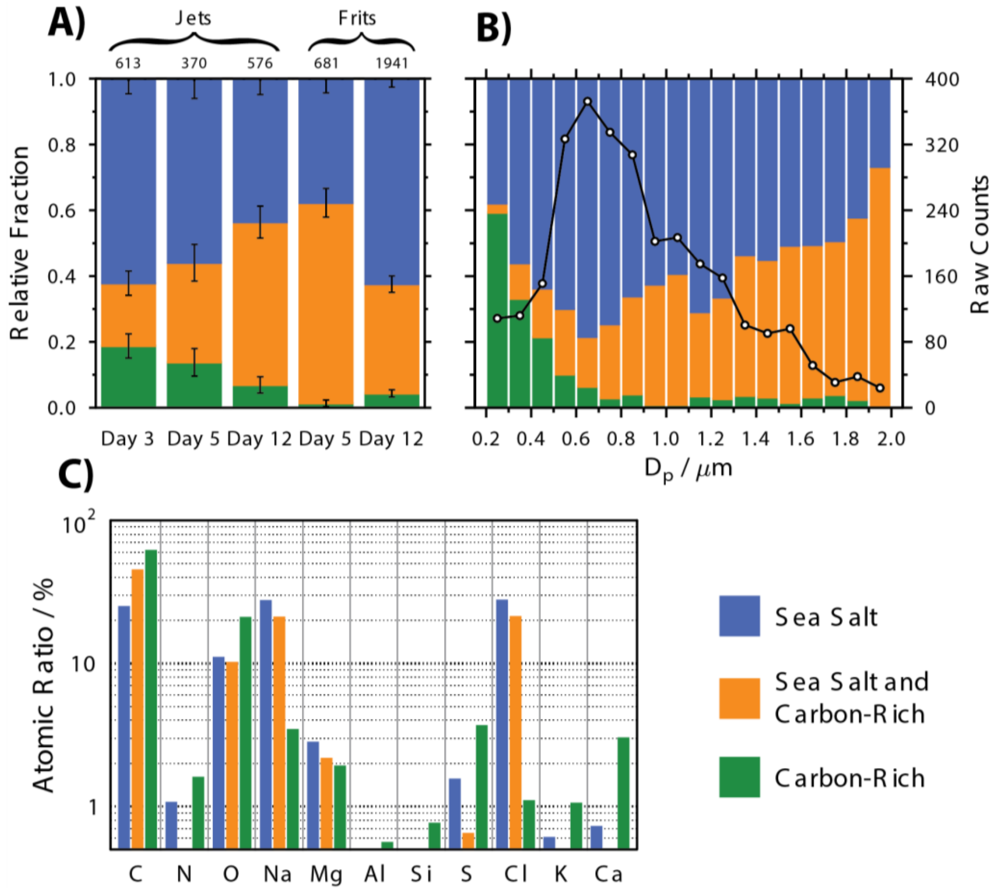

Figure S3: SSA particle classification based on atomic ratio. Results from a *k*-mean cluster analysis for 4,181 investigated particles aerosolized from experiment Tpseu using computer controlled scanning electron microscopy and energy dispersive analysis of X-rays (CCSEM/EDX). Individual particles aerosolized using jets or frits were clustered into three groups, *i*) carbon-rich, *ii*) sea salt and carbon-rich and *iii*) organic-free sea salt shown as green, orange and blue colors, respectively. **A** Mixing state analysis of SSA particles generated after 3, 5 and 12 days of mesocosm age. The number of particles analyzed in each sample is indicated above the bar graph. **B** Size distribution of each particle cluster derived from all samples with the particle numbers analyzed in each size bin are given. The circle equivalent diameter of the imaged particles is given as D_p. **C** Average atomic ratios of the three particle clusters.

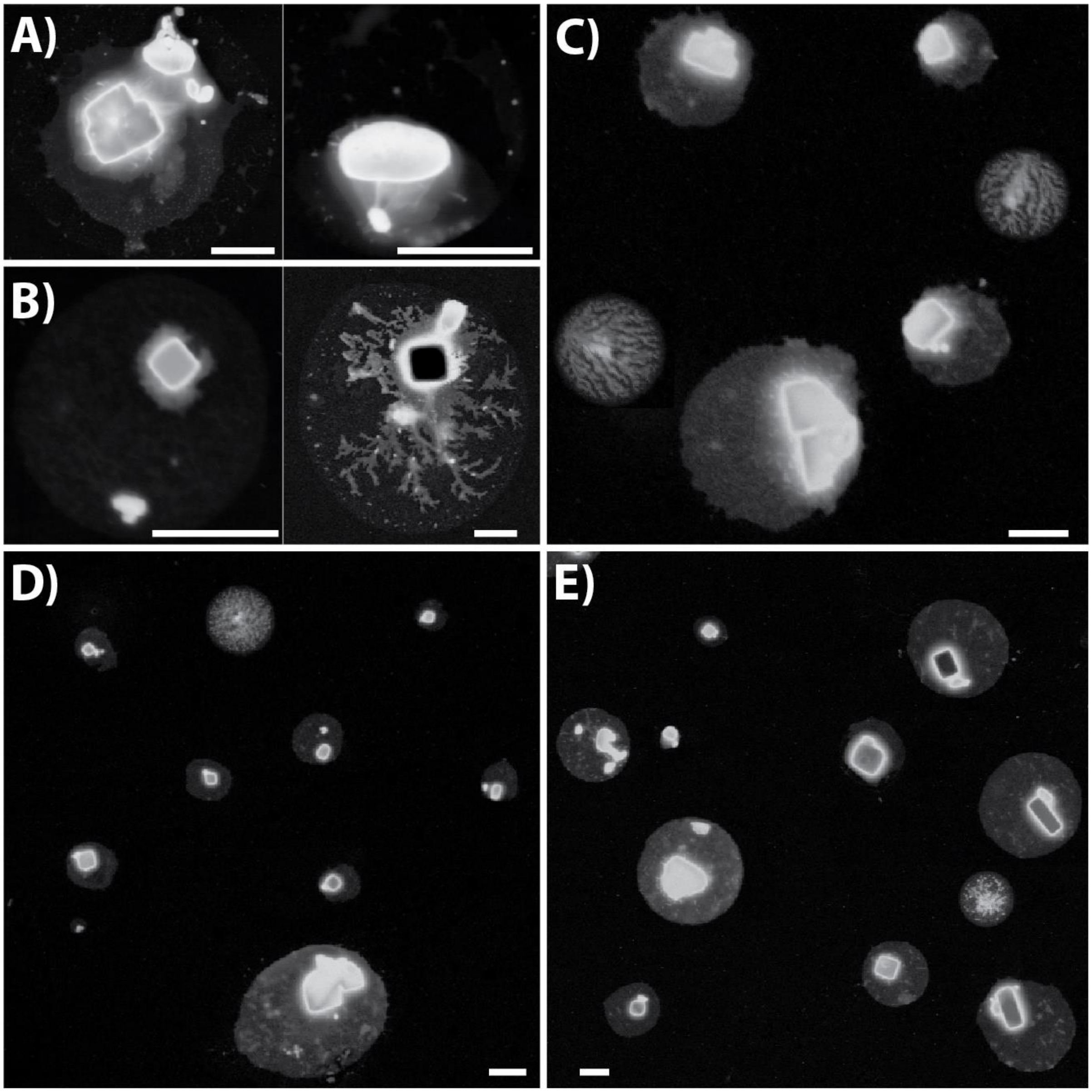

Figure S4. Representative SEM images of SSA particles from experiment Tpseu using STEM detector. SSA particles aerosolized using jets are shown in A), B) and D) after 3, 5, and 12 days of mesocosm age, respectively. SSA particles aerosolized using frits are shown in C) and E) after 5 and 12 days of mesocosm age, respectively. The scale bar for all images is 1 µm.

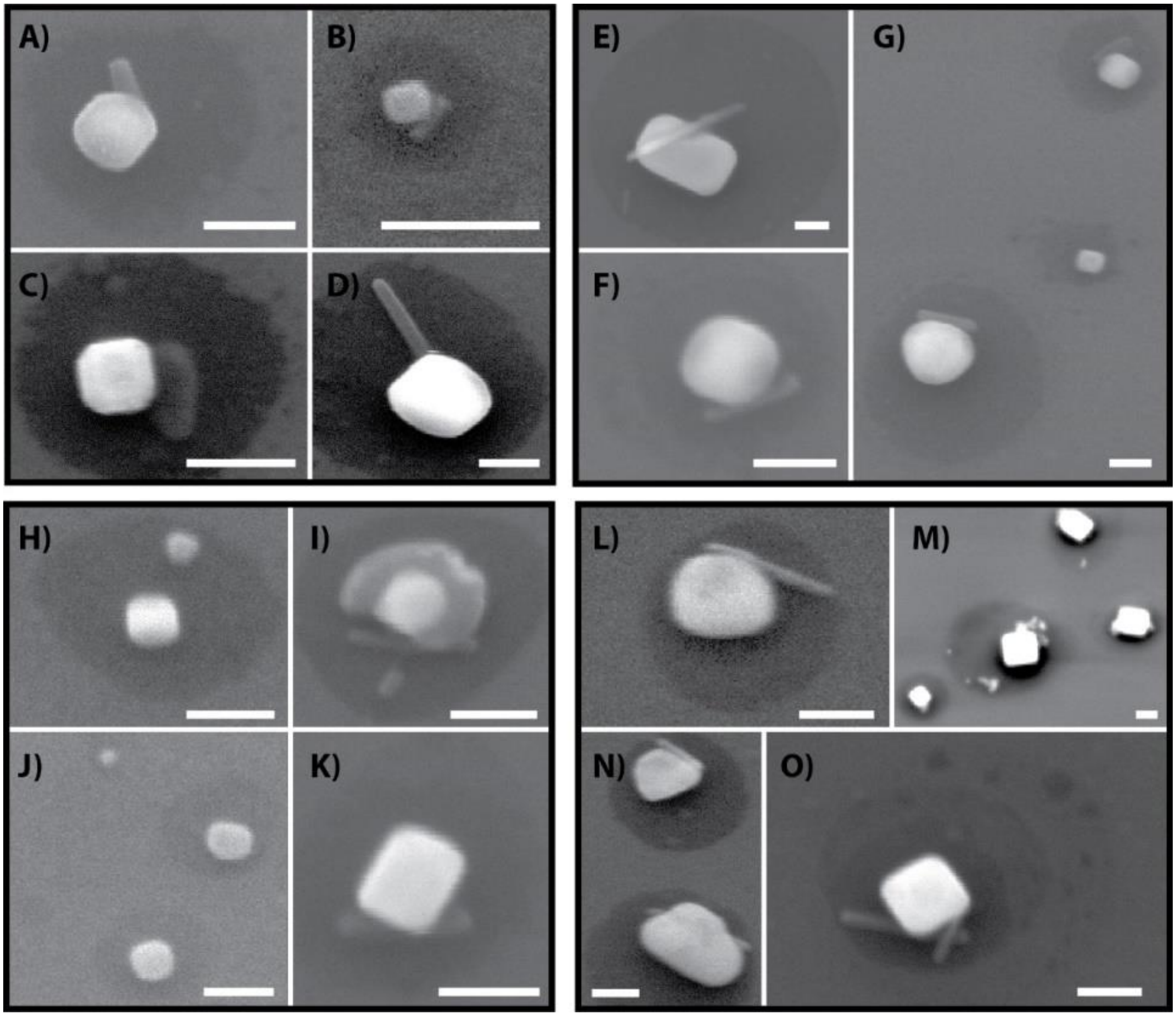

Figure S5. Representative SEM images of SSA particles using SE detector. Images show particles generated using jets. The name and days after the start of the experiment are Gbac, 10 days (A-D), Ehux, 1 day (E and F) and 12 days (G), Natom, 1 day (H and J) and 12 days (I and K), and NSW-1, 0 days (L and N), 3 days (M) and 8 days (O). The scale bar for all images is 0.3 µm.

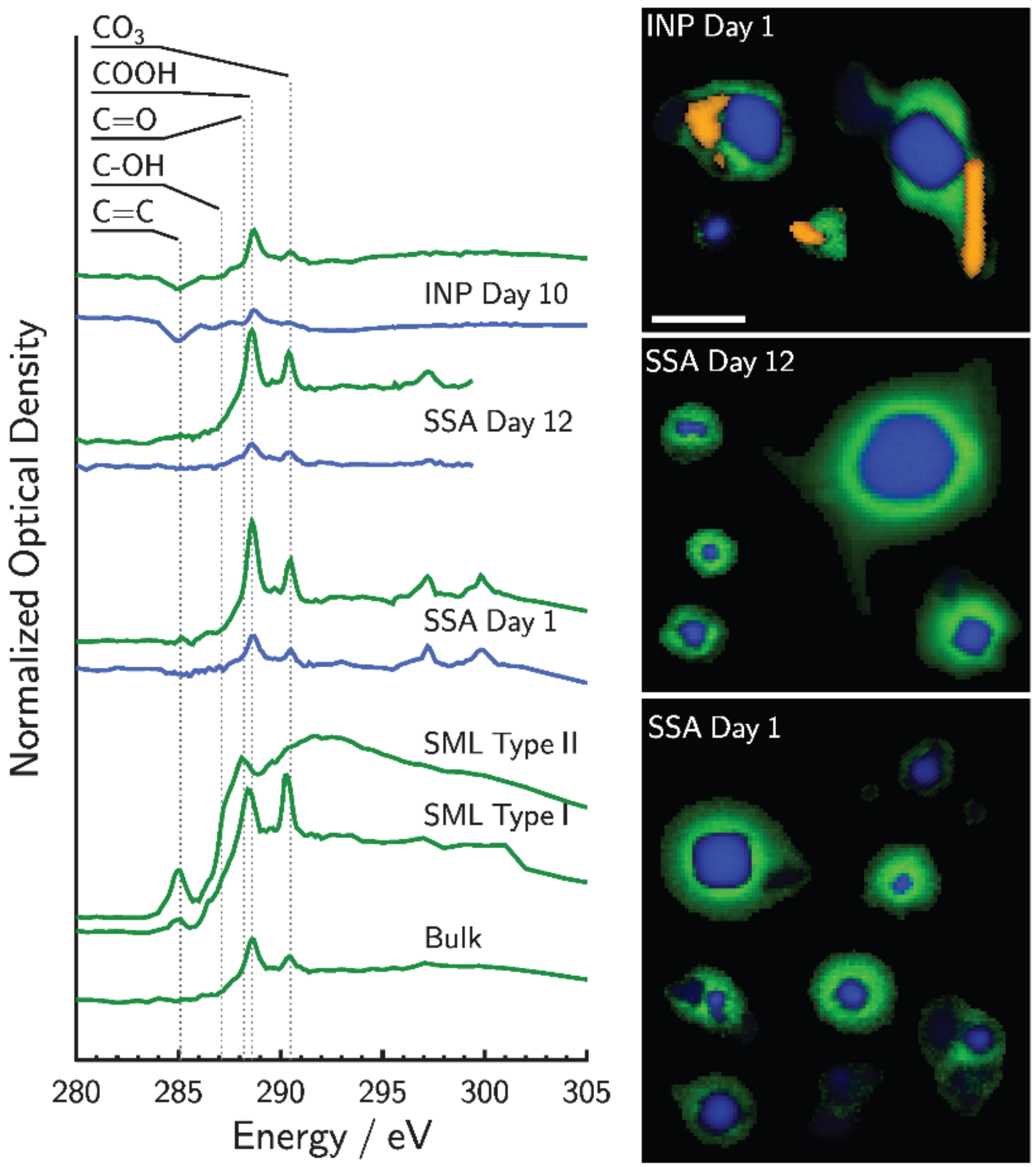

Figure S6. Carbon X-ray spectro-microscopy of water borne and aerosolized particles from experiment Natom. Carbon NEXAFS spectra and corresponding false color X-ray component images of jet generated SSA particles from the mesocosm experiment Natom in which the phytoplankton *N. atomus* was grown over time. When present, orange colors indicate calcium inclusions in SSA particles. Reference spectra of organic material from bulk water and the surface microlayer (SML) were taken from axenic unialgal cultures of *N. atomus*. Spectra were normalized to their total area of optical density versus X-ray energy. Labeled carbon functionalities are carbonate (CO_3), carboxyl (COOH), ketone (C=O), and carbon-carbon double bond (C=C) appearing at 290.5, 288.6, 288.2 and 285.1 eV, respectively. Images of both ice nucleating particles and those particles that did not nucleate ice are included. The day after phytoplankton growth started is indicated. The scale bar is 1 μm and applies to all images.

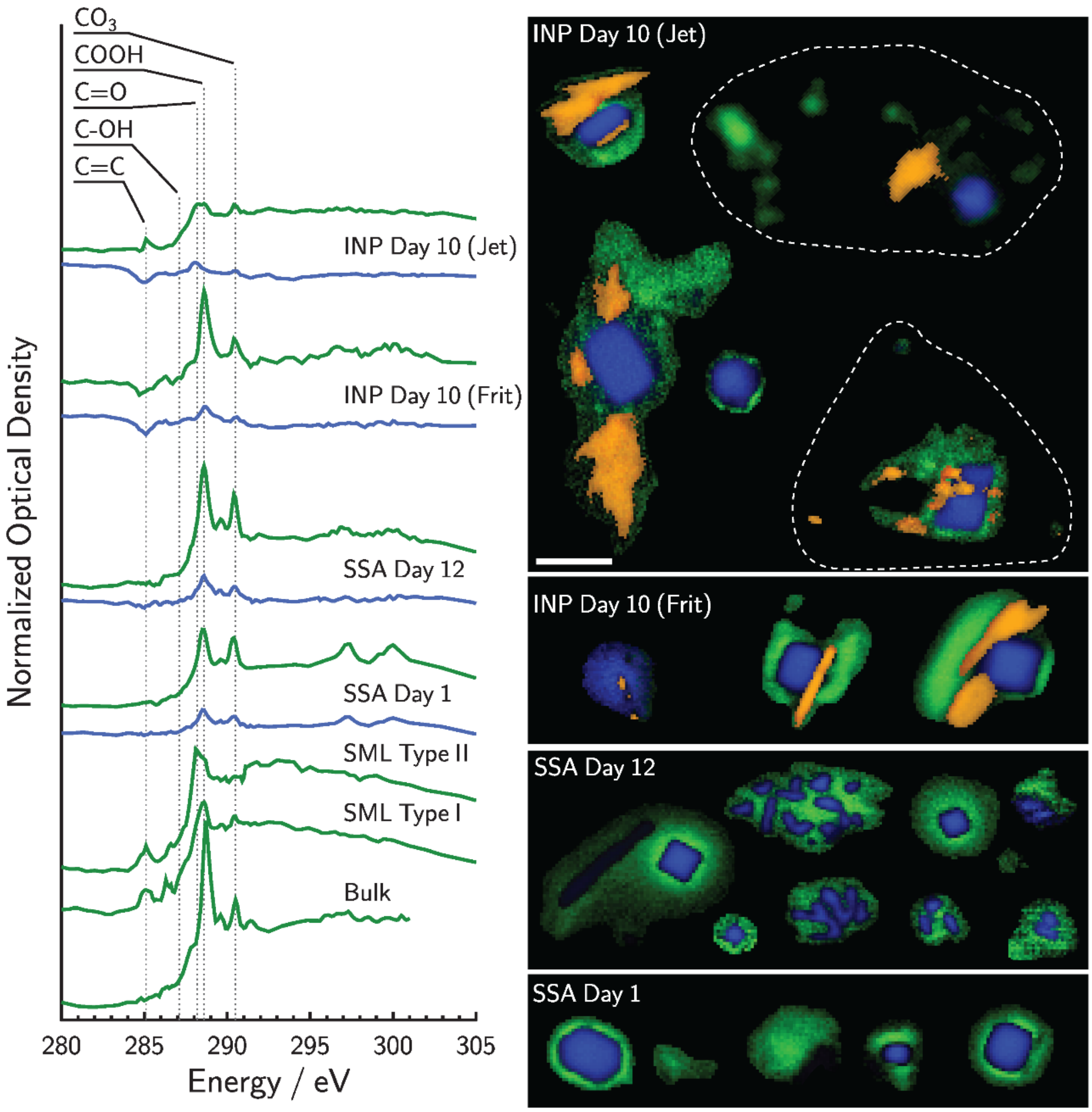

Figure. S7. Carbon X-ray spectro-microscopy of water borne and aerosolized particles from experiment Ehux. Carbon NEXAFS spectra and corresponding false color X-ray component images of jets and frits generated SSA particles from the mesocosm experiment Ehux in which the phytoplankton *E. huxleyi* was grown over time. Reference spectra of OM from bulk water and surface microlayer (SML) were taken from axenic unialgal cultures of *E. huxleyi*. All other labels and lines are the same as Fig. S6.

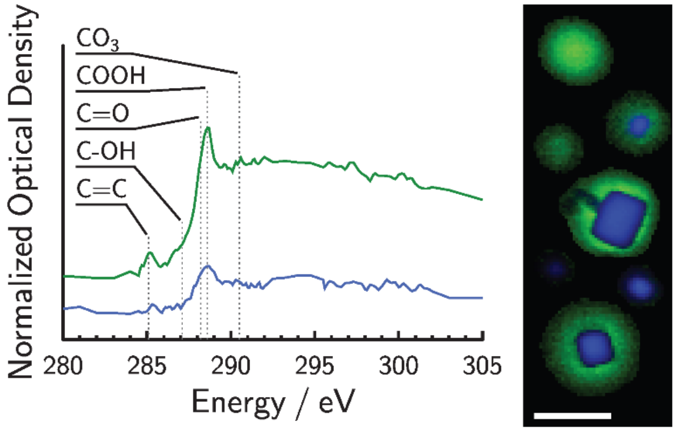

Figure S8. Carbon X-ray spectro-microscopy of water borne and aerosolized particles from experiment Gbac. Carbon NEXAFS spectra and corresponding false color X-ray component images of jets generated SSA particles from the mesocosm experiment Gbac collected after 4 days of growth. All other labels and lines are the same as Fig. S6.

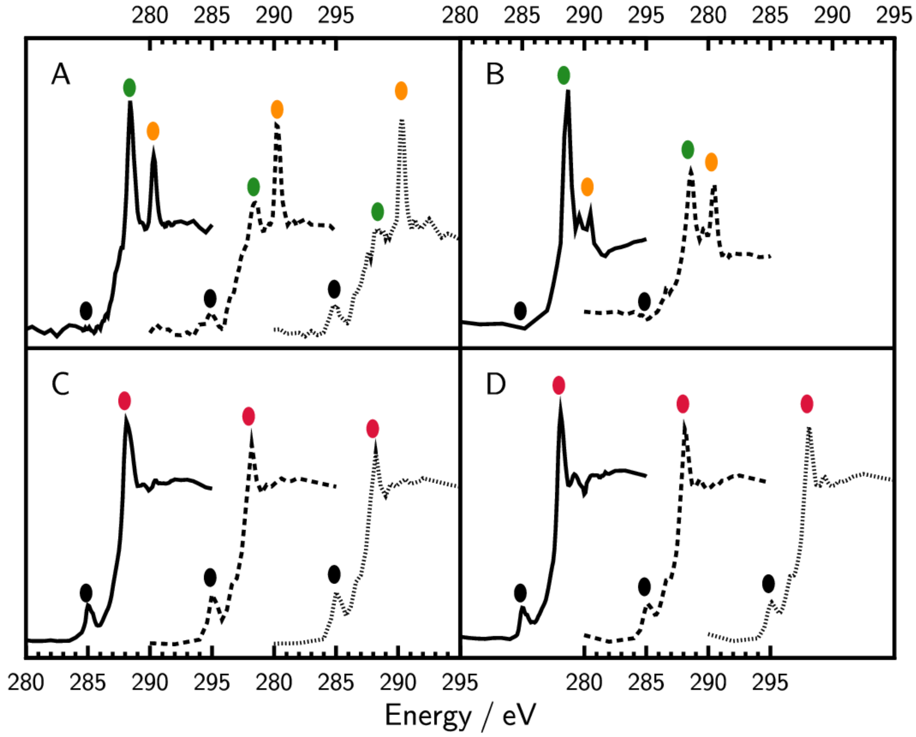

Figure S9. X-ray damage analysis of different organic components in SSA. Beam damage assessment of 4 types of organic carbon spectra A) SML Type I, B) particles from the NatSW mesocosm, C) SML type II, D) xanthan gum. Solid lines indicate spectra acquired during the first X-ray exposure, while the dashed and dotted lines indicate spectra acquired sequentially on the same particles, i.e., two and three times the radiation exposure, respectively. Black, red, green, and orange ovals indicate peaks at 285.1 (C=C), 288.2 (C=O), 288.6 (COOH) and 290.5 eV (CO3), respectively.

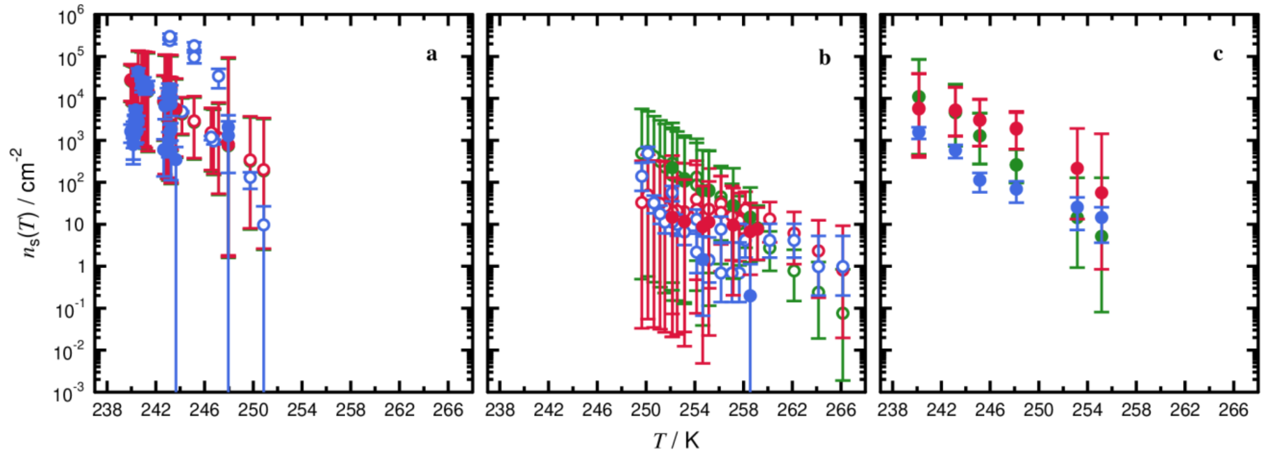

Figure S10. Comparison between simulated and experimentally derived n_s values for sea spray aerosol (SSA) particles. Continuous flow diffusion chamber (CFDC), ice spectrometer (IS), and the multiple orifice uniform distribution impactor-droplet freezing technique (MOUDI-DFT) are shown in a, b and c, respectively. Filled symbols indicate when SSA were generated from a laboratory breaking wave channel (LBWC), and open symbols were generated in a marine aerosol referent tank (MART). Blue symbols and error bars are from DeMott et al. (*12*). Green and red symbols are n_s derived from our stochastic freezing model (SFM) considering variable and identical surface area, respectively. Error bars on red and green symbols were derived from Poisson fiducial limits at x = 0.999 confidence.

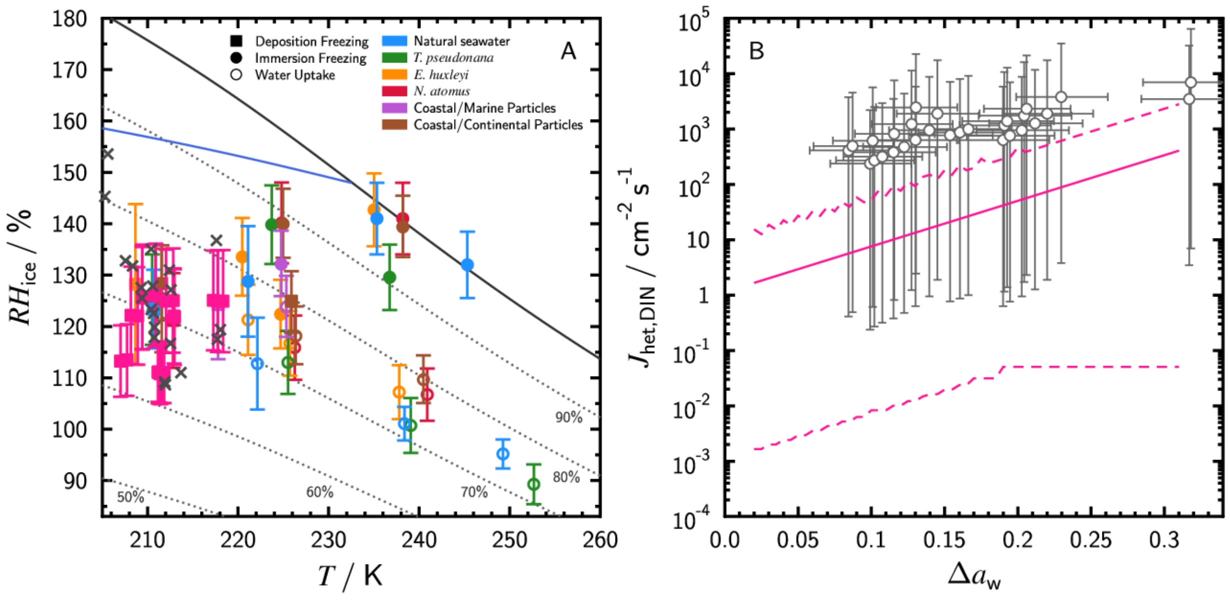

Figure S11. Observation of ice nucleation from SSA particles compared to predictions of deposition ice nucleation (DIN). A) A reproduction of Fig. 1 in the main text where the temperature, T, and relative humidity with respect to ice, RH_{ice}, at which ice formed is plotted. Crosses indicate individual observations of DIN. Pink squares are DIN predicted from the stochastic freezing model (SFM). The error bars are the standard deviation from the SFM using 2000 simulations per data point. All other symbols and lines are the same as in Fig. 1 in the main text. **B)** The heterogeneous ice nucleation rate coefficient for deposition ice nucleation, $J_{\text{het,DIN}}$ as a function of Δa_{w} used to predict the pink squares in A), where $J_{\text{het,DIN}}(\Delta a_{\text{w}}) = 8.2350\Delta a_{\text{w}} + 0.0559$. The upper and lower fiducial limits are shown as dashed lines and reflect a total of 21 freezing events. Symbols and error bars are DIN data from particles stemming from a marine environment taken from Knopf et al. (*45*).

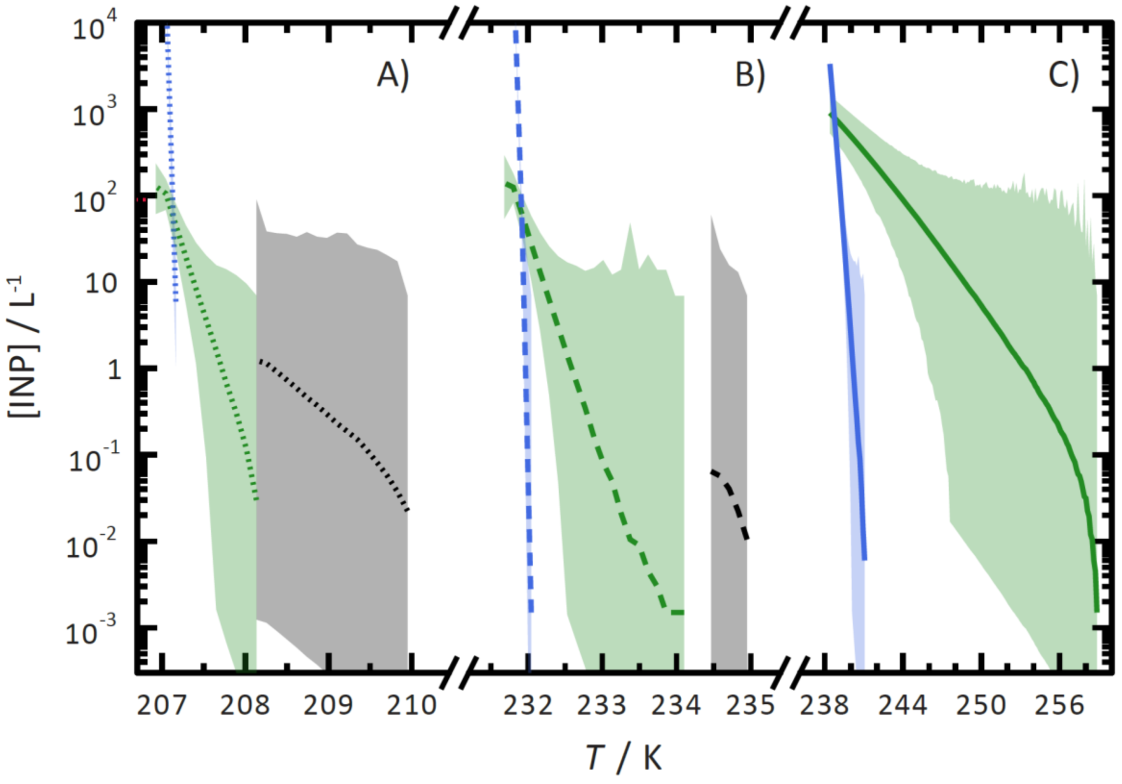

Figure S12. Predicted ice nucleating particle concentrations, [INP], from SSA particles. [INP] from deposition ice nucleation, immersion freezing and homogenous ice nucleation as a function of temperature, *T*, are shown as black, green, and blue colors, respectively. The solid, dashed, and dotted lines are for frost point temperatures, initially at A) 210 K, B) 235 K and C) 260 K, respectively. The shaded areas are Poisson fiducial limits at $x = 0.999$ confidence. Deposition ice nucleation was predicted not to occur for the warmest frost point. Note the breaks in the *T* axis.

Table S1: Observations of single heterogeneous ice nucleation events and water uptake. The mesocosm name, mode of ice nucleation, SSA generation method, mesocosm age in days, temperature, T, relative humidity with respect to ice, RH_{ice}, relative humidity with respect to water, RH, and the sample area, A are given. The water activity shift, Δa_w, is calculated from T and RH. The time difference, Δt, is the time for T to decrease at a rate of 0.1 K min^{-1} from the frost point, when RH_{ice} = 100%, to the T when nucleation was observed.

Name	Mode	SSA Method	Age / d	T / K	RH_{ice} / %	RH / %	A / cm^2	Δa_w	Δt / min
Tpseu	DIN[a]	Jet[d]	12	210.4	135.1	77.1	1.1×10^{-4}	0.201	21.9
				210.4	123.3	70.4		0.134	15.2
				210.7	117.9	67.4		0.102	12.0
	IMF[b]			223.8	139.2	87.2		0.245	27.3
				223.7	139.8	87.5		0.249	27.6
				223.7	140.4	87.9		0.253	28.0
				236.7	130.5	91.5		0.213	24.5
				236.8	128.0	89.9		0.197	22.7
				236.7	130.3	91.5		0.213	24.4
	Wat[c]			225.5	112.8	71.7		0.081	–
				225.5	113.3	72.0		0.085	–
				225.5	112.9	71.8		0.083	–
				239.0	101.2	72.6		0.009	–
				239.6	95.3	68.7		-0.034	–
				238.6	106.0	75.8		0.044	–
				252.7	89.3	73.1		-0.088	–
Ehux	DIN	Jet	5	208.4	131.8	74.3	2.3×10^{-4}	0.179	19.7
				207.6	132.8	74.5		0.184	20.1
	IMF			225.1	124.3	78.7		0.154	18.1
				225.1	118.0	74.7		0.114	13.7
	Wat			225.0	119.3	75.5		0.122	–
				227.1	117.6	75.7		0.113	–
	DIN	Frit[e]	5	209.3	127.6	78.9	1.5×10^{-4}	0.222	17.5
				209.4	125.6	71.2		0.145	16.4
	IMF			224.3	125.4	78.9		0.160	18.7
				224.4	122.0	76.8		0.138	16.4
	Wat			225.2	113.2	71.6		0.082	–
	DIN	Jet	10	212.0	108.7	62.6	1.4×10^{-4}	0.050	6.1
				211.9	109.2	62.9		0.053	6.5
	IMF			220.6	130.6	79.8		0.187	21.3
				220.3	136.6	83.4		0.224	24.9
				234.9	142.6	98.4		0.294	32.3
				235.1	142.8	98.8		0.297	32.5
	Wat			221.1	122.6	75.2		0.138	–
				221.1	120.0	73.7		0.123	–
				237.8	107.2	76.0		0.051	–
	DIN	Frit	10	205.6	153.6	85.2	8.0×10^{-4}	0.298	30.0
				205.3	145.3	80.4		0.251	26.0

Table S1 continued:

Name	Mode	SSA Method	Age / d	T / K	RH_{ice} / %	RH / %	A / cm^2	Δa_w	Δt / min
Natom	DIN	Jet	10	212.4	131.0	75.7	2.2×10^{-4}	0.179	20.0
				212.6	127.1	73.5		0.157	17.8
	IMF			223.3	152.0	94.9		0.325	34.5
				224.8	134.6	85.1		0.219	24.7
	Wat			225.2	119.6	75.8		0.124	–
	DIN	Frit	10	212.5	116.7	67.5	1.5×10^{-4}	0.097	11.4
				213.7	111.0	64.7		0.064	7.8
	IMF			225.6	137.1	87.2		0.236	26.4
				225.6	137.7	87.6		0.240	26.8
				238.2	140.5	100.0		0.288	31.8
	Wat			227.3	112.4	72.5		0.080	–
				240.9	106.7	77.9		0.049	–
NatSW	DIN	Jet	8	210.6	127.9	73.0	1.3×10^{-4}	0.159	17.9
				210.7	122.6	70.0		0.128	14.8
				210.8	119.9	68.5		0.113	13.2
	IMF			221.7	118.9	73.3		0.116	13.9
				220.4	140.3	85.7		0.247	27.1
				221.2	128.1	78.7		0.173	19.9
				235.6	136.4	94.7		0.252	28.4
				235.2	144.2	99.7		0.305	33.4
				235.2	142.7	98.8		0.296	32.4
				245.6	130.8	100.0		0.236	26.6
				245.3	131.2	100.0		0.238	26.9
				245.0	131.7	100.0		0.240	27.2
	Wat			222.2	110.8	68.6		0.067	–
				222.5	107.7	66.8		0.048	–
				221.3	126.3	77.6		0.161	–
				222.6	107.3	66.6		0.045	–
				238.4	100.5	71.7		0.004	–
				238.3	101.7	72.5		0.013	–
				249.9	92.1	73.4		-0.063	–
				249.4	96.8	76.7		-0.026	–
				248.5	96.8	76.1		-0.025	–

Table S1 continued:

Name	Mode	SSA Method	Age / d	T / K	RH_{ice} / %	RH / %	A / cm^2	Δa_w	Δt / min
SI_Oct	DIN	–	–	211.2	132.5	75.9	3.0×10^{-4}	0.186	20.6
				212.4	113.7	65.7		0.079	9.5
				211.2	130.9	75.1		0.178	19.7
	IMF			225.4	135.4	86.0		0.225	25.3
				224.6	146.4	92.4		0.293	31.7
				224.9	139.8	88.4		0.252	27.9
				224.9	138.5	87.6		0.244	27.1
				238.1	140.7	100.0		0.289	31.9
				238.4	137.8	98.2		0.269	30.0
				238.2	139.6	99.3		0.281	31.2
	Wat			226.7	116.6	74.8		0.106	–
				226.4	118.4	75.8		0.118	–
				226.2	119.2	76.2		0.123	–
				226.2	118.5	75.7		0.118	–
				240.4	111.0	80.7		0.080	–
				240.8	106.2	77.5		0.045	–
				240.2	111.9	81.1		0.086	–
BP_Oct	DIN	–	–	211.4	131.9	75.7	7.7×10^{-5}	0.183	20.3
				211.5	126.9	72.9		0.155	17.5
				211.8	126.4	72.7		0.152	17.2
				226.0	122.4	78.1		0.143	16.9
				226.0	125.2	79.9		0.161	18.8
				225.8	126.6	80.6		0.169	19.7
SI_July	DIN	–	–	217.6	136.7	81.8	1.3×10^{-4}	0.220	24.3
				218.1	119.4	71.6		0.116	13.8
				217.7	117.5	70.3		0.104	12.5
	IMF			225.4	133.0	84.5		0.210	23.8
				224.4	131.4	82.7		0.197	22.6
				224.5	132.0	83.2		0.202	23.0
	Wat			226.0	125.2	79.9		0.161	–
				224.7	125.9	79.5		0.164	–
				225.3	120.4	76.3		0.129	–

[a]Deposition ice nucleation. [b]Immersion freezing. [c]Water uptake. [d]SSA generated from water jets. [e]SSA generated from glass frits.

REFERENCES AND NOTES

1. E. R. Lewis, S. E. Schwartz, *Sea Salt Aerosol Production: Mechanisms, Methods, Measurements and Models* (American Geophysical Union, 2004).

2. K. S. Carslaw, L. A. Lee, C. L. Reddington, K. J. Pringle, A. Rap, P. M. Forster, G. W. Mann, D. V. Spracklen, M. T. Woodhouse, L. A. Regayre, J. R. Pierce, Large contribution of natural aerosols to uncertainty in indirect forcing. *Nature* **503**, 67–71 (2013).

3. K. Tsigaridis, N. Daskalakis, M. Kanakidou, P. J. Adams, P. Artaxo, R. Bahadur, Y. Balkanski, S. E. Bauer, N. Bellouin, A. Benedetti, T. Bergman, T. K. Berntsen, J. P. Beukes, H. Bian, K. S. Carslaw, M. Chin, G. Curci, T. Diehl, R. C. Easter, S. J. Ghan, S. L. Gong, A. Hodzic, C. R. Hoyle, T. Iversen, S. Jathar, J. L. Jimenez, J. W. Kaiser, A. Kirkevåg, D. Koch, H. Kokkola, Y. H. Lee, G. Lin, X. Liu, G. Luo, X. Ma, G. W. Mann, N. Mihalopoulos, J. J. Morcrette, J.F. Müller, G. Myhre, S. Myriokefalitakis, N. L. Ng, D. O'Donnell, J. E. Penner, L. Pozzoli, K. J. Pringle, L. M. Russell, M. Schulz, J. Sciare, Ø. Seland, D. T. Shindell, S. Sillman, R. B. Skeie, D. Spracklen, T. Stavrakou, S. D. Steenrod, T. Takemura, P. Tiitta, S. Tilmes, H. Tost, T. van Noije, P. G. van Zyl, K. von Salzen, F. Yu, Z. Wang, Z. Wang, R. A. Zaveri, H. Zhang, K. Zhang, Q. Zhang, X. Zhang, The AeroCom evaluation and intercomparison of organic aerosol in global models. *Atmos. Chem. Phys.* **14**, 10845–10895 (2014).

4. T. Storelvmo, Aerosol effects on climate via mixed-phase and ice clouds. *Annu. Rev. Earth Pl. Sc.* **45**, 199–222 (2017).

5. A. M. Fridlind, A. S. Ackerman, in *Mixed-Phase Clouds: Observation and Modeling*, C. Andronache, Ed. (Elsevier, 2018), chap. 7, pp. 153–183.

6. M. D. Zelinka, S. A. Klein, Y. Qin, T. A. Myers, Evaluating climate models' cloud feedbacks against expert judgment. *J. Geophys. Res. Atmos.* **127**, e2021JD035198 (2022).

7. P. A. Alpert, J. Y. Aller, D. A. Knopf, Ice nucleation from aqueous NaCl droplets with and without marine diatoms. *Atmos. Chem. Phys.* **11**, 5539–5555 (2011).

8. P. A. Alpert, J. Y. Aller, D. A. Knopf, Initiation of the ice phase by marine biogenic surfaces in supersaturated gas and supercooled aqueous phases. *Phys. Chem. Chem. Phys.* **13**, 19882–19894 (2011).

9. E. K. Bigg, Ice nucleus concentrations in remote areas. *J. Atmos. Sci.* **30**, 1153–1157 (1973).

10. S. M. Burrows, C. Hoose, U. Pöschl, M. G. Lawrence, Ice nuclei in marine air: Biogenic particles or dust? *Atmos. Chem. Phys.* **13**, 245–267 (2013).

11. J. M. Creamean, J. N. Cross, R. Pickart, L. McRaven, P. Lin, A. Pacini, R. Hanlon, D. G. Schmale, J. Ceniceros, T. Aydell, N. Colombi, E. Bolger, P. J. DeMott, Ice nucleating particles carried from below a phytoplankton bloom to the arctic atmosphere. *Geophys. Res. Lett.* **46**, 8572–8581 (2019).

12. P. J. DeMott, T. C. J. Hill, C. S. McCluskey, K. A. Prather, D. B. Collins, R. C. Sullivan, M. J. Ruppel, R. H. Mason, V. E. Irish, T. Lee, C. Y. Hwang, T. S. Rhee, J. R. Snider, G. R. McMeeking, S. Dhaniyala, E. R. Lewis, J. J. B. Wentzell, J. Abbatt, C. Lee, C. M. Sultana, A. P. Ault, J. L. Axson, M. Diaz Martinez, I. Venero, G. Santos-Figueroa, M. D. Stokes, G. B. Deane, O. L. Mayol-Bracero, V. H. Grassian, T. H. Bertram, A. K. Bertram, B. F. Moffett, G. D. Franc, Sea spray aerosol as a unique source of ice nucleating particles. *Proc. Natl. Acad. Sci. U.S.A.* **113**, 5797–5803 (2016).

13. V. E. Irish, S. J. Hanna, Y. Xi, M. Boyer, E. Polishchuk, M. Ahmed, J. Chen, J. P. D. Abbatt, M. Gosselin, R. Chang, L. A. Miller, A. K. Bertram, Revisiting properties and concentrations of ice-nucleating particles in the sea surface microlayer and bulk seawater in the Canadian Arctic during summer. *Atmos. Chem. Phys.* **19**, 7775–7787 (2019).

14. D. A. Knopf, P. A. Alpert, A water activity based model of heterogeneous ice nucleation kinetics for freezing of water and aqueous solution droplets. *Faraday Discuss.* **165**, 513–534 (2013).

15. D. A. Knopf, P. A. Alpert, B. Wang, The role of organic aerosol in atmospheric ice nucleation: A review. *ACS Earth Space Chem.* **2**, 168–202 (2018).

16. D. A. Knopf, P. A. Alpert, B. Wang, J. Y. Aller, Stimulation of ice nucleation by marine diatoms. *Nat. Geosci.* **4**, 88–90 (2011).

17. D. A. Knopf, P. A. Alpert, B. Wang, R. E. O'Brien, S. T. Kelly, A. Laskin, M. K. Gilles, R. C. Moffet, Microspectroscopic imaging and characterization of individually identified ice nucleating particles from a case field study. *J. Geophys. Res. Atmos.* **119**, 10,365–10,381 (2014).

18. L. A. Ladino, J. D. Yakobi-Hancock, W. P. Kilthau, R. H. Mason, M. Si, J. Li, L. A. Miller, C. L. Schiller, J.A. Huffman, J. Y. Aller, D. A. Knopf, A. K. Bertram, J. P. D. Abbatt, Addressing the ice nucleating abilities of marine aerosol: A combination of deposition mode laboratory and field measurements. *Atmos. Environ.* **132**, 1–10 (2016).

19. C. S. McCluskey, T. C. J. Hill, C. M. Sultana, O. Laskina, J. Trueblood, M. V. Santander, C. M. Beall, J. M. Michaud, S. M. Kreidenweis, K. A. Prather, V. Grassian, P. J. DeMott, A mesocosm double feature: Insights into the chemical makeup of marine ice nucleating particles. *J. Atmos. Sci.* **75**, 2405–2423 (2018).

20. C. S. McCluskey, J. Ovadnevaite, M. Rinaldi, J. Atkinson, F. Belosi, D. Ceburnis, S. Marullo, T. C. J. Hill, U. Lohmann, Z. A. Kanji, C. O'Dowd, S. M. Kreidenweis, P. J. DeMott, Marine and terrestrial organic ice-nucleating particles in pristine marine to continentally influenced northeast atlantic air masses. *J. Geophys. Res. Atmos.* **123**, 6196–6212 (2018).

21. K. A. Prather, T. H. Bertram, V. H. Grassian, G. B. Deane, M. D. Stokes, P. J. DeMott, L. I. Aluwihare, B. P. Palenik, F. Azam, J. H. Seinfeld, R. C. Moffet, M. J. Molina, C. D. Cappa, F. M. Geiger, G. C. Roberts, L. M. Russell, A. P. Ault, J. Baltrusaitis, D. B. Collins, C. E. Corrigan, L. A. Cuadra-Rodriguez, C. J. Ebben, S. D. Forestieri, T. L. Guasco, S. P. Hersey, M. J. Kim, W. F. Lambert, R. L. Modini, W. Mui, B. E. Pedler, M. J. Ruppel, O. S. Ryder, N. G. Schoepp, R. C. Sullivan, D. Zhao, Bringing the ocean into the laboratory to probe the chemical complexity of sea spray aerosol. *Proc. Natl. Acad. Sci. U.S.A.* **110**, 7550–7555 (2013).

22. G. P. Schill, M. A. Tolbert, Heterogeneous ice nucleation on simulated sea-spray aerosol using raman microscopy. *J. Phys. Chem. C* **118**, 29234–29241 (2014).

23. R. C. Schnell, G. Vali, Freezing nuclei in marine waters. *Tellus* **27**, 321–323 (1975).

24. B. Wang, A. Laskin, T. Roedel, M. K. Gilles, R. C. Moffet, A. V. Tivanski, D. A. Knopf, Heterogeneous ice nucleation and water uptake by field-collected atmospheric particles below 273 K. *J. Geophys. Res. Atmos.* **117**, D00V19 (2012).

25. E. K. Wilbourn, D. C. O. Thornton, C. Ott, J. Graff, P. K. Quinn, T. S. Bates, R. Betha, L. M. Russell, M. J. Behrenfeld, S. D. Brooks, Ice nucleation by marine aerosols over the north atlantic ocean in late spring. *J. Geophys. Res. Atmos.* **125**, e2019JD030913 (2020).

26. T. W. Wilson, L. A. Ladino, P. A. Alpert, M. N. Breckels, I. M. Brooks, J. Browse, S. M. Burrows, K. S. Carslaw, J. A. Huffman, C. Judd, W. P. Kilthau, R. H. Mason, G. McFiggans, L. A. Miller, J. J. Nájera, E. Polishchuk, S. Rae, C. L. Schiller, M. Si, J. V. Temprado, T. F. Whale, J. P. S. Wong, O. Wurl, J. D. Yakobi-Hancock, J. P. D. Abbatt, J. Y. Aller, A. K. Bertram, D. A. Knopf, B. J. Murray, A marine biogenic source of atmospheric ice-nucleating particles. *Nature* **525**, 234–238 (2015).

27. M. J. Wolf, A. Coe, L. A. Dove, M. A. Zawadowicz, K. Dooley, S. J. Biller, Y. Zhang, S. W. Chisholm, D. J. Cziczo, Investigating the heterogeneous ice nucleation of sea spray aerosols using Prochlorococcus as a model source of marine organic matter. *Environ. Sci. Technol.* **53**, 1139–1149 (2019).

28. R. Wagner, L. Ickes, A. K. Bertram, N. Els, E. Gorokhova, O. Möhler, B. J. Murray, N. S. Umo, M. E. Salter, Heterogeneous ice nucleation ability of aerosol particles generated from Arctic sea surface microlayer and surface seawater samples at cirrus temperatures. *Atmos. Chem. Phys.* **21**, 13903–13930 (2021).

29. P. Roy, L. E. Mael, T. C. J. Hill, L. Mehndiratta, G. Peiker, M. L. House, P. J. DeMott, V. H. Grassian, C. S. Dutcher, Ice nucleating activity and residual particle morphology of bulk seawater and sea surface microlayer. *ACS Earth Space Chem.* **5**, 1916–1928 (2021).

30. A. P. Ault, R. C. Moffet, J. Baltrusaitis, D. B. Collins, M. J. Ruppel, L. A. Cuadra-Rodriguez, D. Zhao, T. L. Guasco, C. J. Ebben, F. M. Geiger, T. H. Bertram, K. A. Prather, V. H. Grassian, Size-dependent changes in sea spray aerosol composition and properties with different seawater conditions. *Environ. Sci. Technol.* **47**, 5603–5612 (2013).

31. A. Laskin, R. C. Moffet, M. K. Gilles, J. D. Fast, R. A. Zaveri, B. Wang, P. Nigge, J. Shutthanandan, Tropospheric chemistry of internally mixed sea salt and organic particles: Surprising reactivity of NaCl with weak organic acids. *J. Geophys. Res. Atmos.* **117**, D15302 (2012).

32. L. N. Hawkins, L. M. Russell, Polysaccharides, proteins, and phytoplankton fragments: Four chemically distinct types of marine primary organic aerosol classified by single particle spectromicroscopy. *Adv. Meteorol.* **2010**, 612132 (2010).

33. J. Boese, A. Osanna, C. Jacobsen, J. Kirz, Carbon edge XANES spectroscopy of amino acids and peptides. *J. Electron Spectrosc.* **85**, 9–15 (1997).

34. A. Braun, S. Wirick, A. Kubátová, B. S. Mun, F. E. Huggins, Photochemically induced decarboxylation in diesel soot extracts. *Atmos. Environ.* **40**, 5837–5844 (2006).

35. P. A. Alpert, W. P. Kilthau, D. W. Bothe, J. A. C. Radway, J. Y. Aller, D. A. Knopf, The influence of marine microbial activities on aerosol production: A laboratory mesocosm study. *J. Geophys. Res. Atmos.* **120**, 8841–8860 (2015).

36. P. A. Alpert, D. A. Knopf, Analysis of isothermal and cooling-rate-dependent immersion freezing by a unifying stochastic ice nucleation model. *Atmos. Chem. Phys.* **16**, 2083–2107 (2016).

37. M. E. Wise, K. J. Baustian, T. Koop, M. A. Freedman, E. J. Jensen, M. A. Tolbert, Depositional ice nucleation onto crystalline hydrated NaCl particles: A new mechanism for ice formation in the troposphere. *Atmos. Chem. Phys.* **12**, 1121–1134 (2012).

38. G. C. Cornwell, C. S. McCluskey, E. J. T. Levin, K. J. Suski, P. J. DeMott, S. M. Kreidenweis, K. A. Prather, Direct online mass spectrometry measurements of ice nucleating particles at a california coastal site. *J. Geophys. Res. Atmos.* **124**, 12157–12172 (2019).

39. D. J. Cziczo, K. D. Froyd, C. Hoose, E. J. Jensen, M. Diao, M. A. Zondlo, J. B. Smith, C. H. Twohy, D. M. Murphy, Clarifying the dominant sources and mechanisms of cirrus cloud formation. *Science* **340**, 1320–1324 (2013).

40. N. Hiranuma, S. D. Brooks, R. C. Moffet, A. Glen, A. Laskin, M. K. Gilles, P. Liu, A. M. Macdonald, J. W. Strapp, G. M. McFarquhar, Chemical characterization of individual particles and residuals of cloud droplets and ice crystals collected on board research aircraft in the ISDAC 2008 study. *J. Geophys. Res. Atmos.* **118**, 6564–6579 (2013).

41. S. China, P. A. Alpert, B. Zhang, S. Schum, K. Dzepina, K. Wright, R. C. Owen, P. Fialho, L. R. Mazzoleni, C. Mazzoleni, D. A. Knopf, Ice cloud formation potential by free tropospheric particles from long-range transport over the Northern Atlantic Ocean. *J. Geophys. Res.* **122**, 3065–3079 (2017).

42. Y. Yun, J. E. Penner, An evaluation of the potential radiative forcing and climatic impact of marine organic aerosols as heterogeneous ice nuclei. *Geophys. Res. Lett.* **40**, 4121–4126 (2013).

43. C. S. McCluskey, T. C. J. Hill, F. Malfatti, C. M. Sultana, C. Lee, M. V. Santander, C. M. Beall, K. A. Moore, G. C. Cornwell, D. B. Collins, K. A. Prather, T. Jayarathne, E. A. Stone, F. Azam, S. M. Kreidenweis, P. J. DeMott, A dynamic link between ice nucleating particles released in nascent sea spray aerosol and oceanic biological activity during two mesocosm experiments. *J. Atmos. Sci.* **74**, 151–166 (2017).

44. J. Y. Aller, J. A. C. Radway, W. P. Kilthau, D. W. Bothe, T. W. Wilson, R. D. Vaillancourt, P. K. Quinn, D. J. Coffman, B. J. Murray, D. A. Knopf, Size-resolved characterization of the polysaccharidic and proteinaceous components of sea spray aerosol. *Atmos. Environ.* **154**, 331–347 (2017).

45. D. A. Knopf, J. C. Charnawskas, P. Wang, B. Wong, J. M. Tomlin, K. A. Jankowski, M. Fraund, D. P. Veghte, S. China, A. Laskin, R. C. Moffet, M. K. Gilles, J. Y. Aller, M. A. Marcus, S. Raveh-Rubin, J. Wang, Micro-spectroscopic and freezing characterization of ice-nucleating particles collected in the marine boundary layer in the eastern North Atlantic. *Atmos. Chem. Phys.* **22**, 5377–5398 (2022).

46. T. Koop, B. P. Luo, A. Tsias, T. Peter, Water activity as the determinant for homogeneous ice nucleation in aqueous solutions. *Nature* **406**, 611–614 (2000).

47. D. Q. Pham, R. O'Brien, M. Fraund, D. Bonanno, O. Laskina, C. Beall, K. A. Moore, S. Forestieri, X. Wang, C. Lee, C. Sultana, V. Grassian, C. D. Cappa, K. A. Prather, R. C. Moffet, Biological impacts on carbon speciation and morphology of sea spray aerosol. *ACS Earth Space Chem.* **1**, 551–561 (2017).

48. P. A. Alpert, A. Boucly, S. Yang, H. Yang, K. Kilchhofer, Z. Luo, C. Padeste, S. Finizio, M. Ammann, B. Watts, Ice nucleation imaged with x-ray spectro-microscopy. *Environ. Sci. Atmos* **2**, 335–351 (2022).

49. D. A. Knopf, P. A. Alpert, A. Zipori, N. Reicher, Y. Rudich, Stochastic nucleation processes and substrate abundance explain time-dependent freezing in supercooled droplets. *npj Clim. Atmos. Sci.* **3**, 2 (2020).

50. L. T. Cravigan, M. D. Mallet, P. Vaattovaara, M. J. Harvey, C. S. Law, R. L. Modini, L. M. Russell, E. Stelcer, D. D. Cohen, G. Olsen, K. Safi, T. J. Burrell, Z. Ristovski, Sea spray aerosol organic enrichment, water uptake and surface tension effects. *Atmos. Chem. Phys.* **20**, 7955–7977 (2020).

51. T. Koop, A. Kapilashrami, L. T. Molina, M. J. Molina, Phase transitions of sea-salt/water mixtures at low temperatures: Implications for ozone chemistry in the polar marine boundary layer. *J. Geophys. Res. Atmos.* **105**, 26393–26402 (2000).

52. A. D. Estillore, H. S. Morris, V. W. Or, H. D. Lee, M. R. Alves, M. A. Marciano, O. Laskina, Z. Qin, A. V. Tivanski, V. H. Grassian, Linking hygroscopicity and the surface microstructure of model inorganic salts, simple and complex carbohydrates, and authentic sea spray aerosol particles. *Phys. Chem. Chem. Phys.* **19**, 21101–21111 (2017).

53. B. Wang, D. A. Knopf, Heterogeneous ice nucleation on particles composed of humic-like substances impacted by O_3. *J. Geophys. Res.* **116**, D03205 (2011).

54. A. P. Hitchcock, C. Morin, X. Zhang, T. Araki, J. Dynes, H. Stöver, J. Brash, J. R. Lawrence, G. G. Leppard, Soft X-ray spectromicroscopy of biological and synthetic polymer systems. *J. Electron Spectrosc.* **144-147**, 259–269 (2005).

55. G. Polzonetti, C. Battocchio, G. Iucci, M. Dettin, R. Gambaretto, C. di Bello, V. Carravetta, Thin films of a self-assembling peptide on TiO_2 and Au studied by NEXAFS, XPS and IR spectroscopies. *Mat. Sci. Eng. C* **26**, 929–934 (2006).

56. R. C. Moffet, A. V. Tivanski, M. K. Gilles, in *Fundamentals and Applications of Aerosol Spectroscopy*, R. Signorell, J. Reid, Eds. (CRC Press, 2010), chap. 17, pp. 419–462.

57. J. Wingender, T. R. Neu, H.-C. Flemming, in *Microbial Extracellular Polymeric Substances: Characterization, Structure and Function*, J. Wingender, T. R. Neu, H.-C. Flemming, Eds. (Springer Berlin Heidelberg, 1999), pp. 1–19.

58. D. B. Collins, D. F. Zhao, M. J. Ruppel, O. Laskina, J. R. Grandquist, R. L. Modini, M. D. Stokes, L. M. Russell, T. H. Bertram, V. H. Grassian, G. B. Deane, K. A. Prather, Direct aerosol chemical composition measurements to evaluate the physicochemical differences between controlled sea spray aerosol generation schemes. *Atmos. Meas. Tech.* **7**, 3667–3683 (2014).

59. T. Koop, B. Zobrist, Parameterizations for ice nucleation in biological and atmospheric systems. *Phys. Chem. Chem. Phys.* **11**, 10839–10850 (2009).

60. G. C. Cornwell, C. S. McCluskey, P. J. DeMott, K. A. Prather, S. M. Burrows, Development of heterogeneous ice nucleation rate coefficient parameterizations from ambient measurements. *Geophys. Res. Lett.* **48**, e2021GL095359 (2021).

61. G. Saliba, C. L. Chen, S. Lewis, L. M. Russell, L. H. Rivellini, A. K. Y. Lee, P. K. Quinn, T. S. Bates, N. Haëntjens, E. S. Boss, L. Karp-Boss, N. Baetge, C. A. Carlson, M. J. Behrenfeld, Factors driving the seasonal and hourly variability of sea-spray aerosol number in the North Atlantic. *Proc. Natl. Acad. Sci. U.S.A.* **116**, 20309–20314 (2019).

62. M. E. Wise, E. J. Freney, C. A. Tyree, J. O. Allen, S. T. Martin, L. M. Russell, P. R. Buseck, Hygroscopic behavior and liquid-layer composition of aerosol particles generated from natural and artificial seawater. *J. Geophys. Res. Atmos.* **114**, D03201 (2009).

63. C. Misra, M. Singh, S. Shen, C. Sioutas, P. M. Hall, Development and evaluation of a personal cascade impactor sampler (PCIS). *J. Aerosol Sci.* **33**, 1027–1047 (2002).

64. A. F. Stein, R. R. Draxler, G. D. Rolph, B. J. B. Stunder, M. D. Cohen, F. Ngan, NOAA's HYSPLIT atmospheric transport and dispersion modeling system. *B. Am. Meteorol. Soc.* **96**, 2059–2077 (2015).

65. C. Marcolli, B. P. Luo, T. Peter, Mixing of the organic aerosol fractions: Liquids as the thermodynamically stable phases. *J. Phys. Chem. A* **108**, 2216–2224 (2004).

66. B. Wang, R. E. O'Brien, S. T. Kelly, J. E. Shilling, R. C. Moffet, M. K. Gilles, A. Laskin, Reactivity of liquid and semisolid secondary organic carbon with chloride and nitrate in atmospheric aerosols. *J. Phys. Chem. A* **119**, 4498–4508 (2015).

67. K. Benzerara, N. Menguy, P. López-García, T.H. Yoon, J. Kazmierczak, T. Tyliszczak, F. Guyot, G. E. Brown Jr., Nanoscale detection of organic signatures in carbonate microbialites. *Proc. Natl. Acad. Sci. U.S.A.* **103**, 9440–9445 (2006).

68. A. G. Hardie, J. J. Dynes, L. M. Kozak, P. M. Huang, The role of glucose in abiotic humification pathways as catalyzed by birnessite. *J. Mol. Catal. A: Chem.* **308**, 114–126 (2009).

69. T. Koop, B. Luo, U. M. Biermann, P. J. Crutzen, T. Peter, Freezing of HNO_3/H2SO_4/H2O solutions at stratospheric temperatures: Nucleation statistics and experiments. *J. Phys. Chem. A* **101**, 1117–1133 (1997).

70. P. J. Connolly, O. Möhler, P. R. Field, H. Saathoff, R. Burgess, T. Choularton, M. Gallagher, Studies of heterogeneous freezing by three different desert dust samples. *Atmos. Chem. Phys.* **9**, 2805–2824 (2009).

71. R. H. Mason, C. Chou, C. S. McCluskey, E. J. T. Levin, C. L. Schiller, T. C. J. Hill, J. A. Huffman, P. J. DeMott, A. K. Bertram, The micro-orifice uniform deposit impactor–Droplet freezing technique (MOUDI-DFT) for measuring concentrations of ice nucleating particles as a function of size: Improvements and initial validation. *Atmos. Meas. Tech.* **8**, 2449–2462 (2015).

72. V. A. Marple, K. L. Rubow, S. M. Behm, A microorifice uniform deposit impactor (MOUDI): Description, calibration, and use. *Aerosol Sci. Tech.* **14**, 434–446 (1991).

73. M. D. Petters, S. M. Kreidenweis, A single parameter representation of hygroscopic growth and cloud condensation nucleus activity. *Atmos. Chem. Phys.* **7**, 1961–1971 (2007).

74. P. J. DeMott, A. J. Prenni, X. Liu, S. M. Kreidenweis, M. D. Petters, C. H. Twohy, M. S. Richardson, T. Eidhammer, D. C. Rogers, Predicting global atmospheric ice nuclei distributions and their impacts on climate. *Proc. Natl. Acad. Sci. U.S.A.* **107**, 11217–11222 (2010).

75. G. M. McFarquhar, S. Ghan, J. Verlinde, A. Korolev, J. W. Strapp, B. Schmid, J. M. Tomlinson, M. Wolde, S. D. Brooks, D. Cziczo, M. K. Dubey, J. Fan, C. Flynn, I. Gultepe, J. Hubbe, M. K. Gilles, A. Laskin, P. Lawson, W. R. Leaitch, P. Liu, X. Liu, D. Lubin, C. Mazzoleni, A.M. Macdonald, R. C. Moffet, H. Morrison, M. Ovchinnikov, M. D. Shupe, D. D. Turner, S. Xie, A. Zelenyuk, K. Bae, M. Freer, A. Glen, Indirect and semi-direct aerosol campaign. *B. Am. Meteorol. Soc.* **92**, 183–201 (2011).

76. J. Savre, A. M. L. Ekman, Large-eddy simulation of three mixed-phase cloud events during ISDAC: Conditions for persistent heterogeneous ice formation. *J. Geophys. Res. Atmos.* **120**, 7699–7725 (2015).

77. P. Spichtinger, D. J. Cziczo, Impact of heterogeneous ice nuclei on homogeneous freezing events in cirrus clouds. *J. Geophys. Res. Atmos.* **115**, D14208 (2010).
